# Supplementary material for: Durable organic nonlinear optical membranes for thermotolerant lightings and in vivo bioimaging
Source: Nat Commun. 2023 Jul 22;14:4429. doi: 10.1038/s41467-023-40168-2 (PMC10363139; doi:10.1038/s41467-023-40168-2)
Supplement: Supplementary file 1 — Supplementary Information [file 41467_2023_40168_MOESM1_ESM.pdf]

Supplementary Information

Durable organic nonlinear optical membranes for thermotolerant lightings and in vivo bioimaging

Tian Tian[1 #], Yuxuan Fang[1 #], Wenhui Wang[1], Meifang Yang[1], Ying Tan[1], Chuan Xu[2], Shuo Zhang[1], Yuxin Chen[3], Mingyi Xu[4]*, Bin Cai[2]*, Wu-Qiang Wu[1]*

[1]MOE Key Laboratory of Bioinorganic and Synthetic Chemistry, Lehn Institute of Functional Materials, School of Chemistry, Sun Yat-sen University; Guangzhou 510006, P. R. China

[2]Shanghai Key Lab of Modern Optical System, Ministry of Education, University of Shanghai for Science and Technology, Shanghai 200093, China

[3]Instrumental Analysis and Research Center, Sun Yat-sen University; Guangzhou 510275, P. R. China

[4]Guangdong Key Laboratory of Environmental Catalysis and Health Risk Control, School of Environmental Science and Engineering, Institute of Environmental Health and Pollution Control, Guangdong University of Technology, Guangzhou 510006, China

[#]These authors contributed equally to this work.

Email: anasyxmy@foxmail.com ; bullcai@usst.edu.cn; wuwq36@mail.sysu.edu.cn

Inventory of Supplementary Information

Supplementary Fig. 1. Schematic illustration of the molecular structures of HPβCD, DAST and BTCA…………………………………………………… **5**
Supplementary Fig. 2. A 25 cm × 15 cm DAST@HPβCD fibers produced via electrospinning for 30 min……………………………………………….. **6**
Supplementary Table 1. Cost evaluation of fabricating 1 m^2 of DAST@HPβCD fabrics……………………………..……………………………………… **7**
Supplementary Fig. 3. FTIR spectra of HPβCD, BTCA and HPβCD@BTCA samples……………………………………………………………………… **8**
Supplementary Fig. 4. The circular dichroism spectroscopy……………………. **9**
Supplementary Fig. 5. Schematic illustration of the insertion mode of DAST molecule in the cavity of HPβCD molecule……………………………………… **10**
Supplementary Table 2. The ^{1}H NMR results of DAST crystals and DAST@HPβCD composites………………………..………………………… **11**
Supplementary Table 3. The ^{1}H NMR results of HPβCD powders and DAST@HPβCD composites…………………………………………………..… **12**
Supplementary Fig. 6. XRD patterns of DAST crystals, HPβCD powders and DAST@HPβCD fibers……………………………………………………….. **13**
Supplementary Fig. 7. Schematic illustration of the hydrogen bonding interactions and self-assembly process……………………………………………………….. **14**
Supplementary Fig. 8. The SEM image of the hydrogen bonding interactions and self-assembly process……………………………………………………………… **15**
Supplementary Fig. 9. The SEM image of DAST@HPβCD fibers after post-annealing treatment and exposure in high humidity condition………………..… **16**
Supplementary Fig. 10. AFM topography image of the surface of DAST@HPβCD thin film……………………………………………………………………..…… **17**
Supplementary Table 4. Crystallographic data and structure refinement parameters for DAST single crystals…………………...……………………. **18**
Supplementary Fig. 11. The PL spectra of the DAST crystals dissolved in different solvents with different polarity……………………………………………….. **19**
Supplementary Fig. 12. Cyclic voltammetry (CV) curves…………….……… **20**
Supplementary Fig. 13. The absorption and PL spectra of DAST crystal and DAST@HPβCD fibers……………………………………………………….. **21**
Supplementary Fig. 14. Potential energy diagrams…………………………….. **22**
Supplementary Fig. 15. The PLQY measurement of DAST@HPβCD fibers.… **23**
Supplementary Fig. 16. PL intensity of DAST and DAST@HPβCD complex dissolved in DMF solution…………………………………………………. **24**
Supplementary Fig. 17. The PLQY measurement of DAST@HPβCD thin film..**25**
Supplementary Table 5. Summarized 1PEF, 2PEF and 3PEF properties of DAST (powder/nanocrystal) and DAST@HPβCD fibers…………………………..….. **26**
Supplementary Table 6. Summarized PL decay lifetime (τ_a), PLQY, radiative recombination rate and non-radiative recombination rate for DAST crystals and

DAST@HPβCD fibers………………………………………………….……. 27
Supplementary Fig. 18. Temperature-dependent PL decay lifetimes of DAST crystals……………………………………………………………………….. 28
Supplementary Table 7. Summarized temperature-dependent PL decay lifetimes of DAST crystals……………………………………………………………… 29
Supplementary Fig. 19. Temperature-dependent PL decay lifetimes of DAST@HPβCD fibers……………………………………………..………… 30
Supplementary Table 8. Summarized temperature-dependent PL decay lifetimes of DAST@HPβCD fibers…………………………………………………….. 31
Supplementary Fig. 20. TG spectra…………………………………………… 32
Supplementary Fig. 21. DSC spectra…………………………………..………. 33
Supplementary Fig. 22. Time-dependent PLQY evolution of DAST@HPβCD fibers stored in ambient air…………………………………………….……... 34
Supplementary Fig. 23. The photographs under bright field and fluorescence images under UV light of DAST@HPβCD thin film heated at 100 ℃, 200 ℃ and 300 ℃……………………………………………………………………..….. 35
Supplementary Fig. 24. The PLQY of DAST@HPβCD thin film after heating at 300 ℃ for 20 min…………………………………………………………….... 36
Supplementary Fig. 25. Raman spectra…………………...………………… 37
Supplementary Fig. 26. Photographs of DAST@PS fibrous membrane under natural light and UV light………………..……………………………………… 38
Supplementary Fig. 27. The fluctuation of two-photon action cross-section of DAST@HPβCD fibers with the change of excitation wavelength………………. 39
Supplementary Fig. 28. Setup of 3PEF evaluation…………………………..…. 40
Supplementary Fig. 29. The 3PEF images of DAST@HPβCD taken at different excitation power……………………………………………………………… 41
Supplementary Fig. 30. The PL intensity change of DAST@HPβCD fibers before and after being soaked in water for 4000 hours……………………………..... 42
Supplementary Fig. 31. The SHG signals of the reference KDP and DAST@HPβCD fibers soaked in water for 4000 hours……………………….. 43
Supplementary Fig. 32. Time-dependent PL intensity of the DAST@HPβCD fibers upon UV light irradiation for different durations……………………….. 44
Supplementary Fig. 33. The confocal laser scanning microscopy (CLSM) images of DAST@HPβCD fibers soaked in water……………………………………. 45
Supplementary Fig. 34. The CLSM images of *E. coli* being fed with the DAST@HPβCD fibers……………………………………...…………... 46
Supplementary Fig. 35. The CLSM images of DAST@HPβCD-stained *E. coli* taken every second at bright-filed mode………………………………………… 47
Supplementary Fig. 36. The CLSM pseudo-colored images at 1PEF of DAST@HPβCD-stained *E. coli* ……………………………………………… 48
Supplementary Fig. 37. The CLSM pseudo-colored images at 2PEF of DAST@HPβCD-stained *E. coli* ……………………………………………… 49
Supplementary Fig. 38. The CLSM images of DAST@HPβCD fibers immersed in culture solution without *E. coli* inoculation for 96 h………………………… 50

Supplementary Table 9. A summary of the state-of-the-art inorganic transition metal chalcogenides quantum dots/nanocrystals, organic fluorescence materials or organic/inorganic hybrid materials for bioimaging application………………… **51**
Supplementary Fig. 39. The CLMS images of DAST@HPβCD-stained *E. coli* cells which were continuously fed with LB medium for 14 day………………… **53**
Supplementary Fig. 40. The impact of different amounts of DAST@HPβCD fibers (as indicated) on the growth performance of *E. coli*…………………………..… **54**
Supplementary Fig. 41. The CLSM images of 20 mg DAST@HPβCD-stained *E. coli* taken every second using bright-filed mode……………………………….. **55**
Supplementary Fig. 42. The CLSM pseudo-colored images at 1PEF of 20 mg DAST@HPβCD-stained *E. coli* ……………………………………………….. **56**
Supplementary Fig. 43. The CLSM pseudo-colored images at 1PEF of 20 mg DAST@HPβCD-stained *E. coli*………………………………………………… **57**
Supplementary References …………………………………………………… **58**

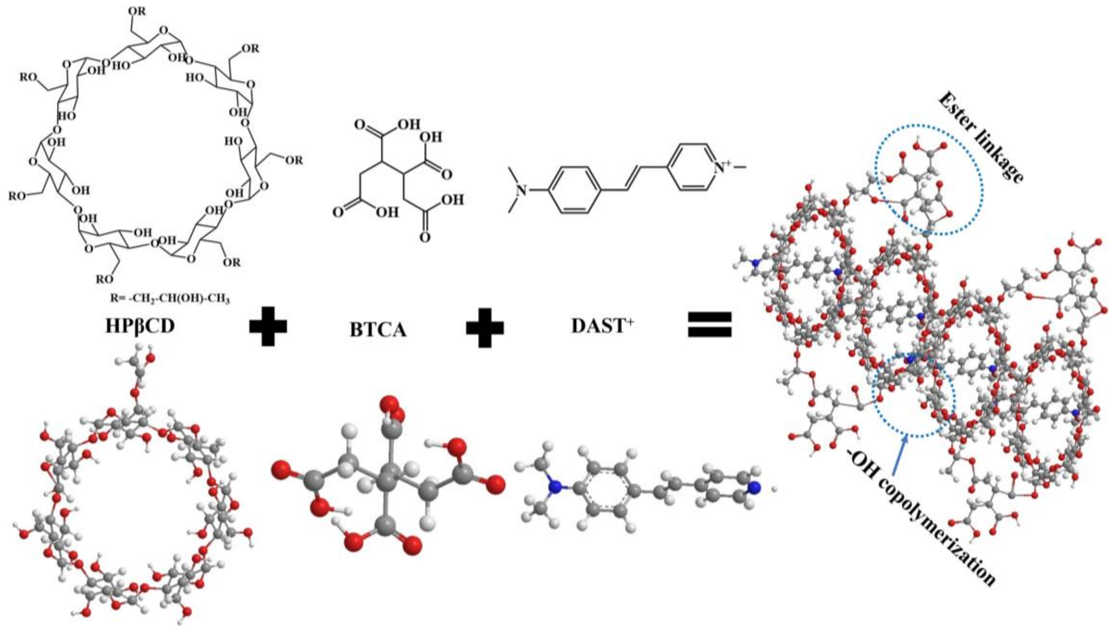

Supplementary Fig. 1. Schematic illustration of the molecular structures of HPβCD, DAST and BTCA, as well as their chemical interactions (i.e. ester linkage and copolymerization) for forming host-guest supramolecular complex.

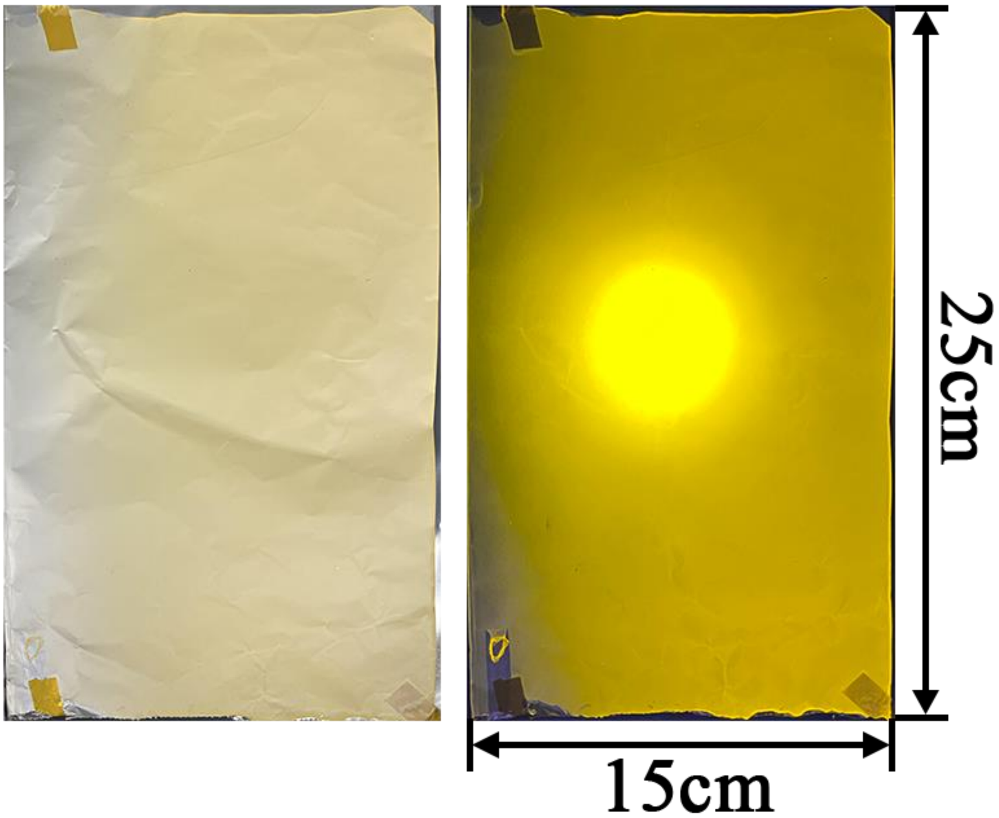

Supplementary Fig. 2. A 25 cm × 15 cm DAST@HPβCD fibers produced via electrospinning for 30 min. Photographs of a DAST@HPβCD fibers prepared via electrospinning for 30 min under (left) natural light and (right) UV light.

Supplementary Table 1. Cost evaluation of fabricating 1 m^2 of DAST@HPβCD fabrics.

DAST@HPβCD **fabrics**	**Ingredients**	**Unit price ($/g)**	**Usage amount (g)**	**Price ($)**
	DAST	14.81 /1	0.0267	0.395
	BTCA	9.31 /25	0.8	0.298
	HPβCD	16.38 /100	8	1.31
	DMF	3.32 /500	26.7	0.177
**Total price**				2.18

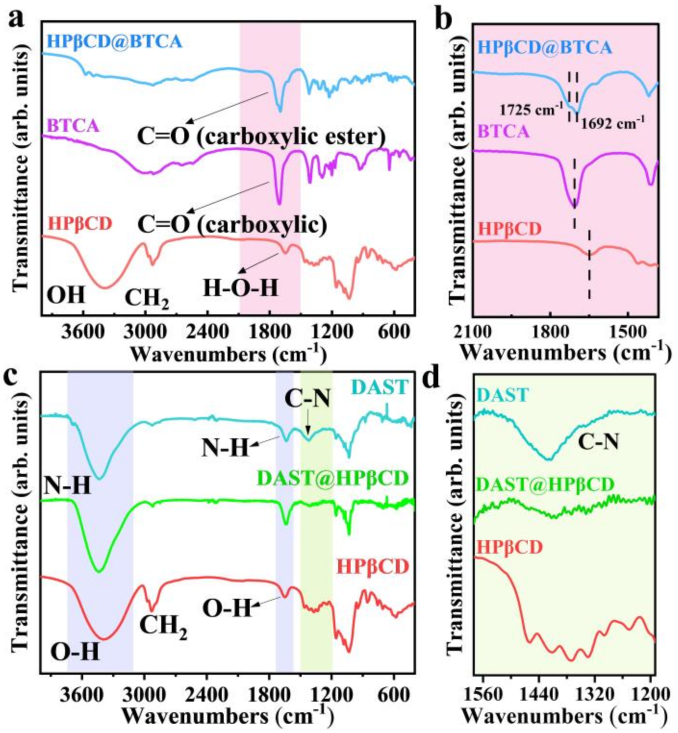

Supplementary Fig. 3. (a) FTIR spectra of HPβCD, BTCA and HPβCD@BTCA samples. (b) The zoom-in view of (a) within a range from 1400 cm^{-1} to 2100 cm^{-1}. (c) FTIR spectra of DAST, DAST@HPβCD and HPβCD samples. (d) The zoom-in view of (c) within a wavenumber range from 1200 cm^{-1} to 1600 cm^{-1}.

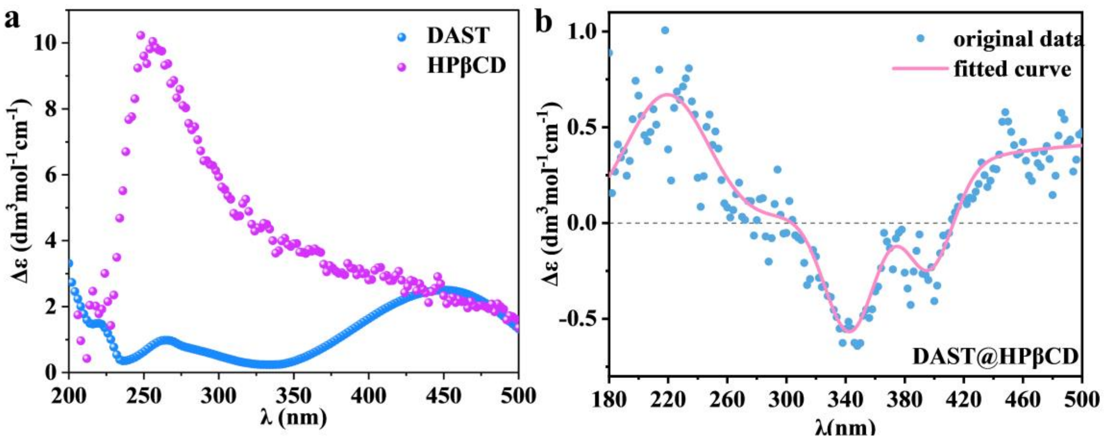

Supplementary Fig. 4. The circular dichroism spectroscopy of DAST crystal, HPβCD powder (a) and DAST@HPβCD composite (b). All the samples were dissolved in water for testing.

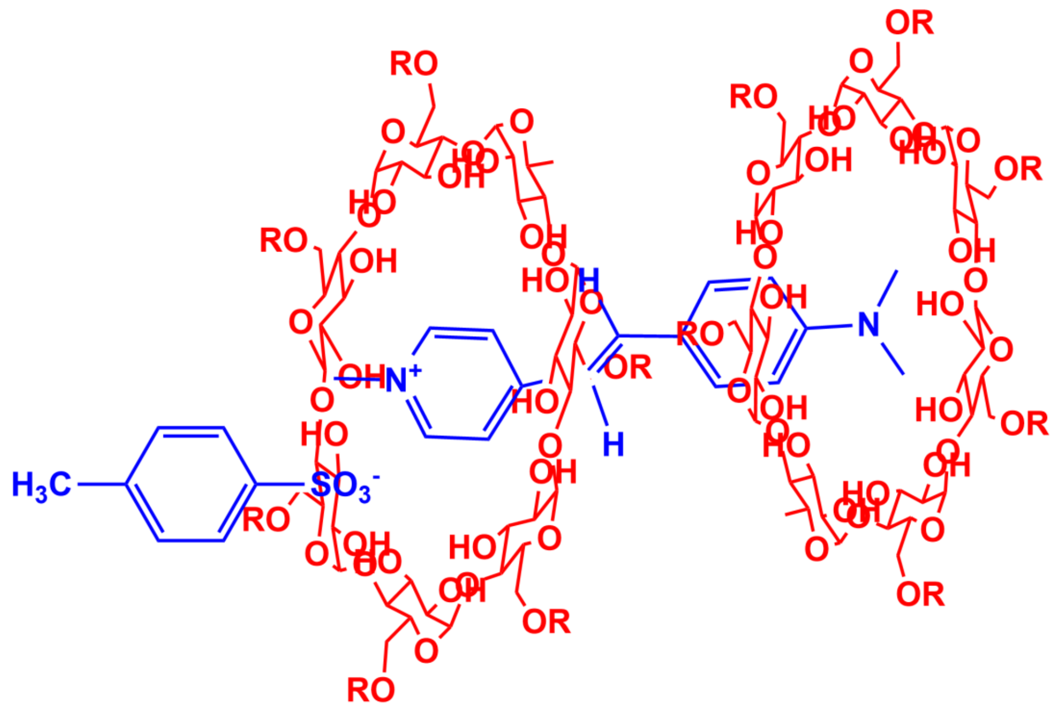

Supplementary Fig. 5. Schematic illustration of the insertion mode of DAST molecule in the cavity of HPβCD molecule.

Supplementary Table 2. The ^{1}H NMR results of DAST crystals and DAST@HPβCD composites. All the samples were dissolved in D_2O for testing.

**Proton**	**DAST (ppm)**	**DAST@HPβCD (ppm)**
**H^1**	3.03018	3.02685
**H^2**	4.17646	4.17329
**H^3**	6.79018	6.80001
**H^4**	7.12260	7.12649
**H^5**	7.59230	7.59185
**H^6**	7.89983	7.89136
**H^7**	8.05314	8.03619
**H^8**	8.41727	8.49227

Supplementary Table 3. The ^{1}H NMR results of HPβCD powders and DAST@HPβCD composites. All the samples were dissolved in D_2O for testing.

Proton	HPβCD (ppm)	DAST@HPβCD (ppm)
**H^1**	5.73001	5.74620
**H^2**	3.31259	3.31153
**H^3**	3.75467	3.74983
**H^4**	3.23283	3.22799
**H^5**	3.56882	3.56382
**H^6**	3.62300	3.61785

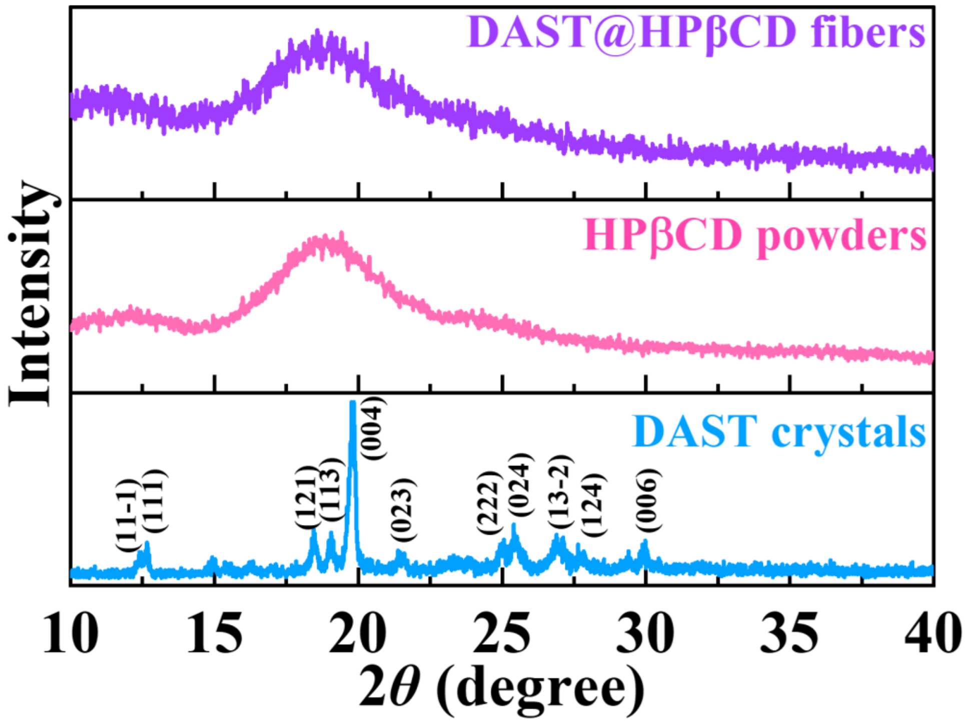

Supplementary Fig. 6. XRD patterns of DAST crystals, HPβCD powders and DAST@HPβCD fibers.

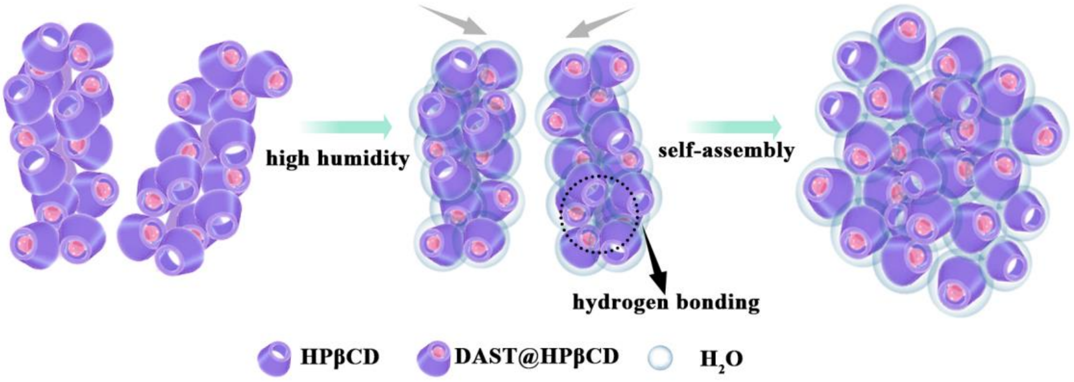

Supplementary Fig. 7. Schematic illustration of the hydrogen bonding interactions and self-assembly process of forming densified DAST@HPβCD network under high humidity condition.

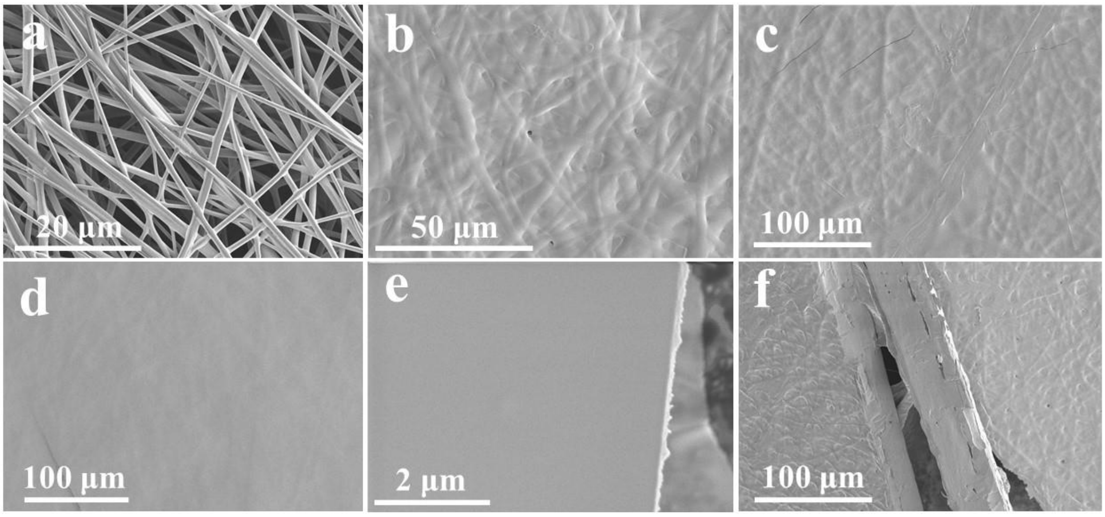

Supplementary Fig. 8. (a) The SEM image of as-prepared DAST@HPβCD fibers. (b-e) The SEM images of the copolymerization process among adjacent fibers to form compact DAST@HPβCD thin films. (f) The cross-sectional SEM image of the copolymerized DAST@HPβCD thin film.

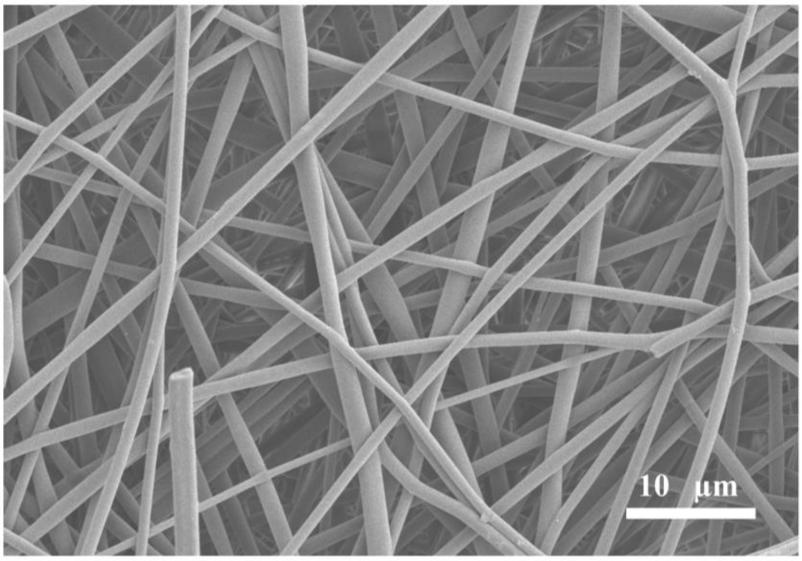

Supplementary Fig. 9. The SEM image of DAST@HPβCD fibers after post-annealing treatment and exposure in high humidity condition.

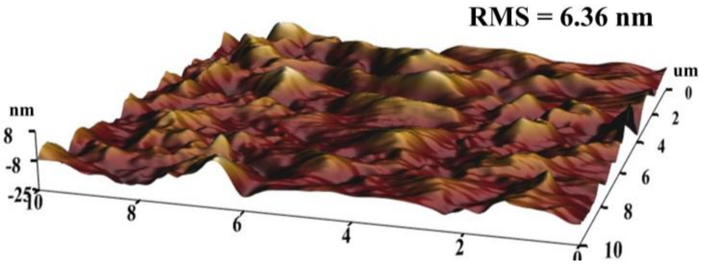

Supplementary Fig. 10. AFM topography image of the surface of DAST@HPβCD thin film.

Supplementary Table 4. Crystallographic data and structure refinement parameters for DAST single crystals.

Identification code	A22011805A0583
Empirical formula	$C_{23}H_{26}N_2O_3S$
Formula weight	410.52
Temperature/K	202.99(10)
Crystal system	monoclinic
Space group	Cc
a/Å	10.3354(2)
b/Å	11.2161(2)
c/Å	17.7840(3)
α/°	90
β/°	92.4450(18)
γ/°	90
Volume/Å^3	2059.70(7)
Z	4
ρ_{calc}g/cm^3	1.324
μ/mm^{-1}	1.614
F(000)	872.0
Crystal size/mm^3	0.19 × 0.09 × 0.05
Radiation	CuKα (λ = 1.54184)
2θ range for data collection/°	9.956 to 148.136
Index ranges	$-12 \leq h \leq 12$, $-13 \leq k \leq 13$, $-22 \leq l \leq 22$
Reflections collected	17288
Independent reflections	4066 [R_{int} = 0.0483, R_{sigma} = 0.0341]
Data/restraints/parameters	4066/2/266
Goodness-of-fit on F^2	1.051
Final R indexes [I>=2σ (I)]	R_1 = 0.0348, wR_2 = 0.0892
Final R indexes [all data]	R_1 = 0.0374, wR_2 = 0.0911
Largest diff. peak/hole / e Å^{-3}	0.22/-0.18
Flack parameter	0.017(11)

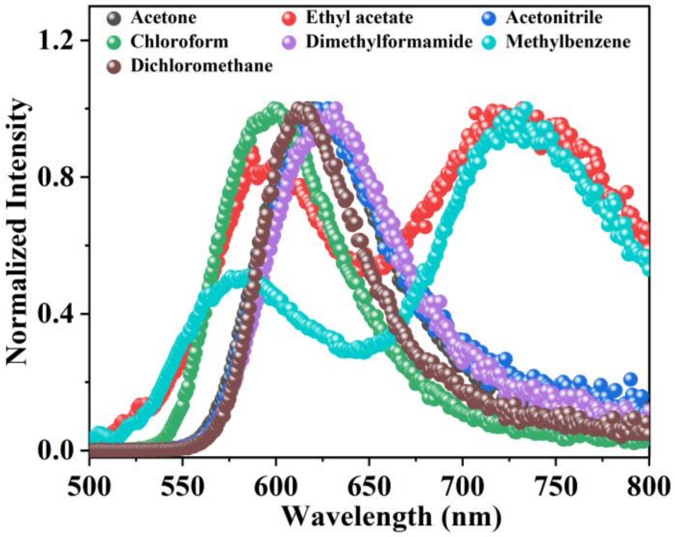

Supplementary Fig. 11. The PL spectra of the DAST crystals dissolved in different solvents with different polarity.

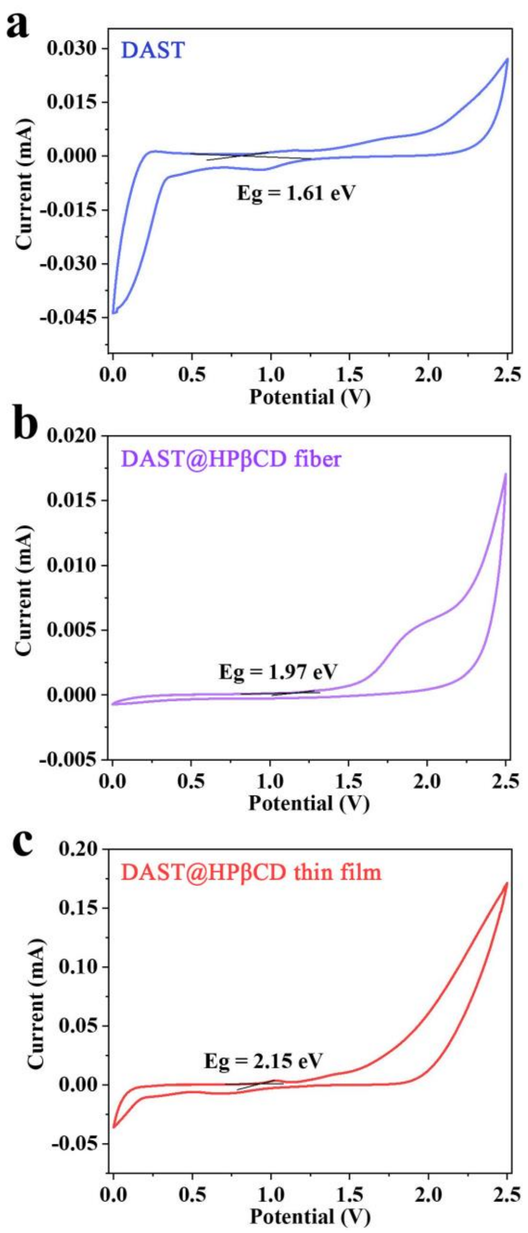

Supplementary Fig. 12. Cyclic voltammetry (CV) curves of (a) DAST crystal, (b) DAST@HPβCD fibers and (c) DAST@HPβCD thin film.

HOMO=-(E_{ox}-$E_{Fc+/Fc}$ + 4.8), LUMO=HOMO + E_g, where E_{ox} is the oxidation potential point, $E_{Fc+/Fc}$ is the oxidation potential of ferrocene. Both of the E_{ox} and $E_{Fc+/Fc}$ are extracted from cyclic voltammetry measurement.

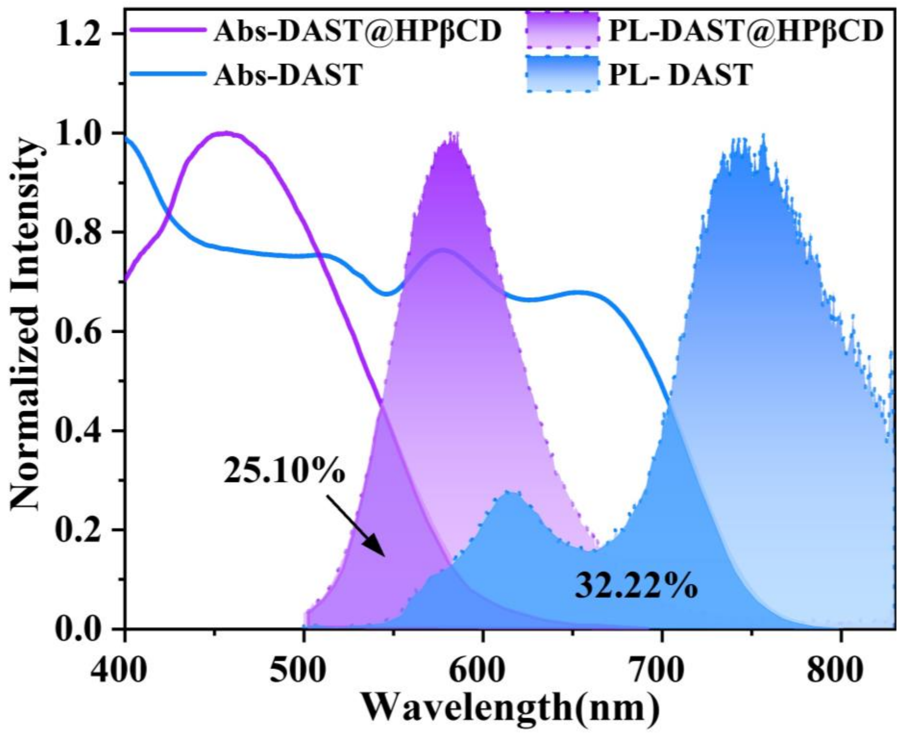

Supplementary Fig. 13. The absorption and PL spectra of DAST crystal (bule color) and DAST@HPβCD fibers (purple color).

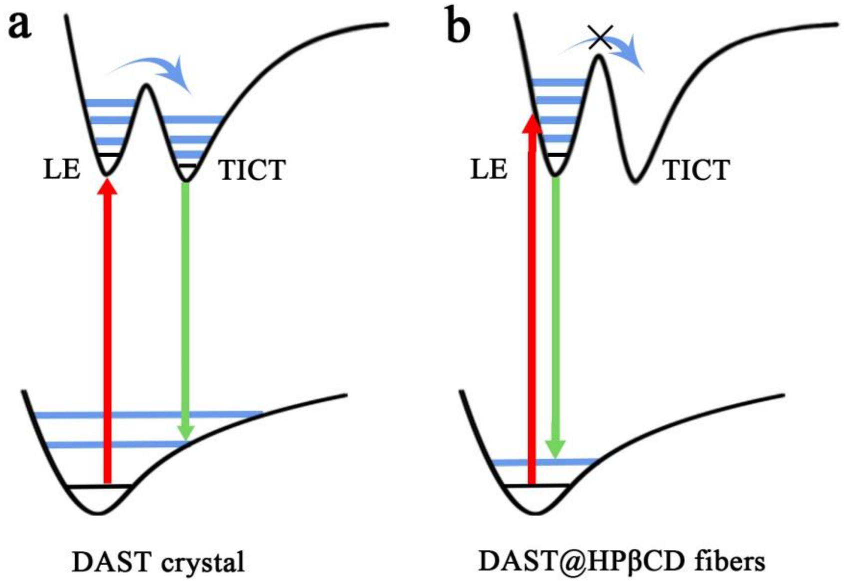

Supplementary Fig. 14. Potential energy diagrams and corresponding excited-state conversion of (a) DAST crystals and (b) DAST@HPβCD fibers.

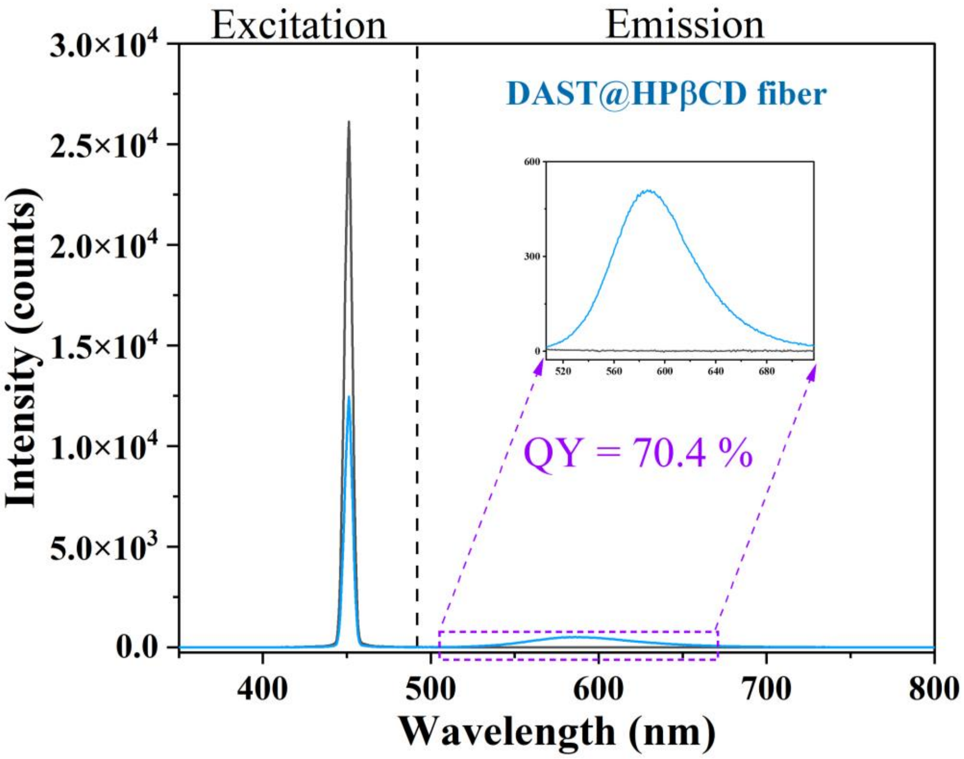

Supplementary Fig. 15. The PLQY measurement of DAST@HPβCD fibers.

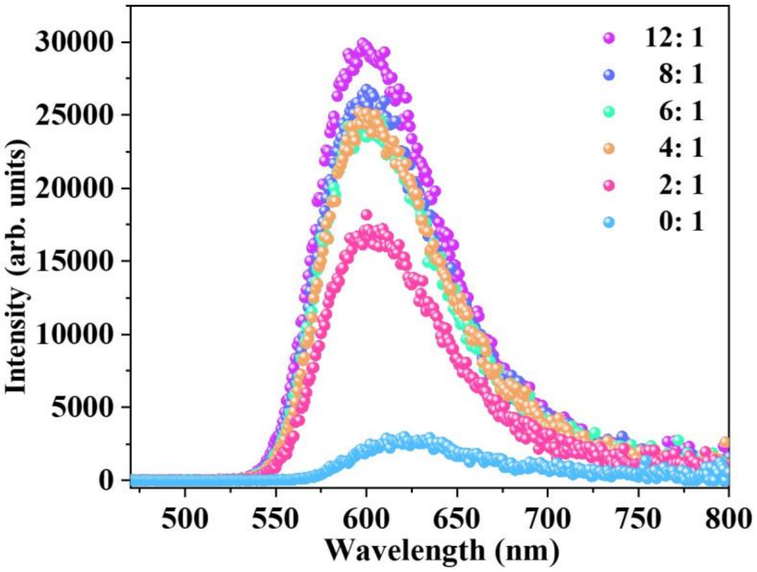

Supplementary Fig. 16. PL intensity of DAST and DAST@HPβCD complex dissolved in DMF solution with different HPβCD: DAST molar ratios as indicated.

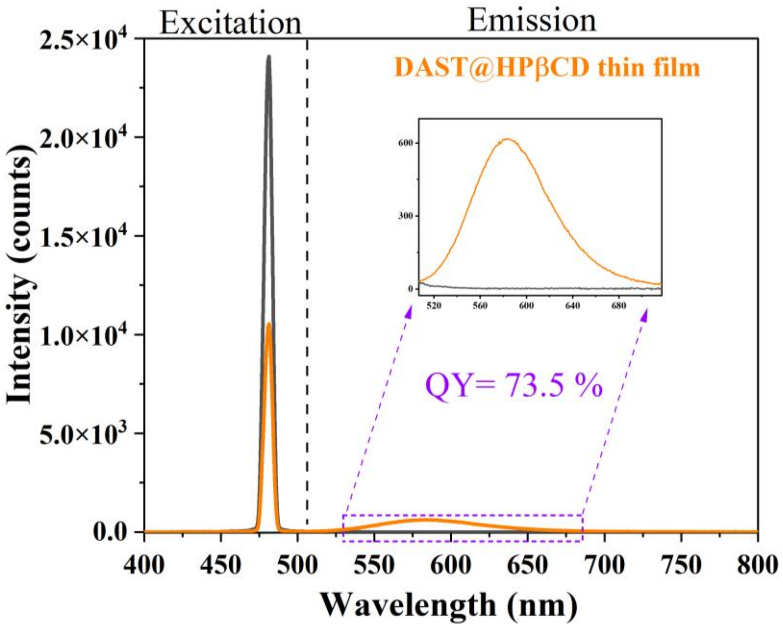

Supplementary Fig. 17. The PLQY measurement of DAST@HPβCD thin film.

Supplementary Table 5. Summarized 1PEF, 2PEF and 3PEF properties of DAST (powder/nanocrystal) and DAST@HPβCD fibers.

**Materials**	**1PEF Emission/Excitation (nm)**	**2PEF Emission/Excitation (nm)**	**3PEF Emission/Excitation (nm)**
**DAST**	615, 746/420[a] (powder)	606/932[1] 585/800[2] (nanocrystal)	N/A
**DAST@HPβCD fibers (this work)**	583/450[b]	578/770[c] 584/1000[d]	585/1590[e]

[a, b] The data is extracted from Supplementary Fig. 13.
[c, d] The data is extracted from Fig. 5b.
[e] The data is extracted from Fig. 5c.

Supplementary Table 6. Summarized PL decay lifetime (τ_a), PLQY, radiative recombination rate and non-radiative recombination rate for DAST crystals and DAST@HPβCD fibers measured at 300 K.

Samples	τ_a (ns)	PLQY (%)	κ_r (10^6/s^1)*	κ_{nr} (10^6/s^1)*
DAST crystals	3.534	0.9	2.54	280.4
DAST@HPβCD fibers	2.999	70.4	234.7	98.7

* The PL decay lifetime, PLQY, radiative recombination rate and non-radiative recombination rate are calculated based on the following equations:

$$PLQY = \kappa_r/(\kappa_{r+}\kappa_{nr}) \quad (1)$$

$$\tau_a = 1/(\kappa_{r+}\kappa_{nr}) \quad (2)$$

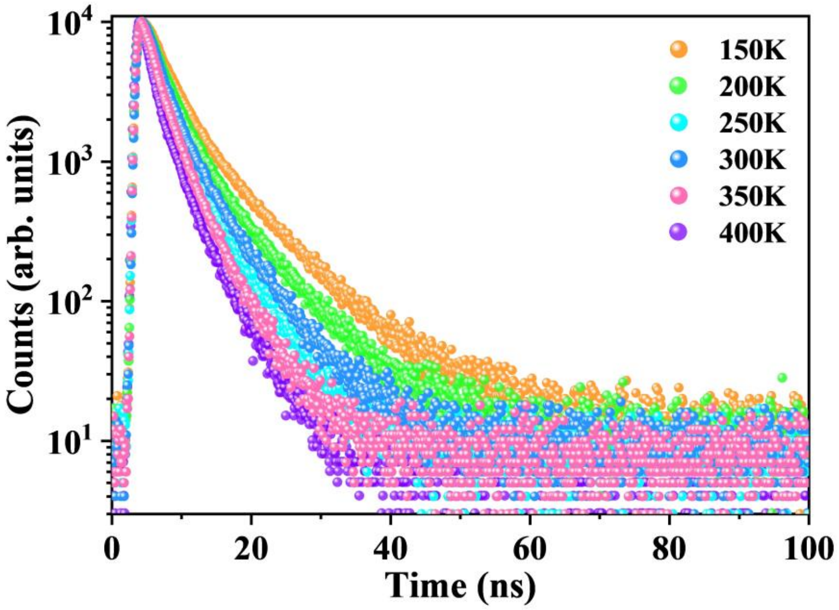

Supplementary Fig. 18. Temperature-dependent PL decay lifetimes of DAST crystals.

Supplementary Table 7. Summarized temperature-dependent PL decay lifetimes of DAST crystals.

**Temperature (K)**	$\boldsymbol{\tau_1}$ **(ns)**	$\mathbf{A_1}$ **(%)**	$\boldsymbol{\tau_2}$ **(ns)**	$\mathbf{A_2}$ **(%)**	$\boldsymbol{\tau_{average}}$ **(ns)**
**150**	3.280	56.24	9.156	43.76	5.851
**200**	2.964	64.35	8.040	35.65	4.774
**250**	2.662	67.30	6.763	32.70	4.003
**300**	2.284	61.49	5.529	38.51	3.534
**350**	2.228	68.33	5.073	31.67	3.129
**400**	1.569	55.74	4.253	44.26	2.757

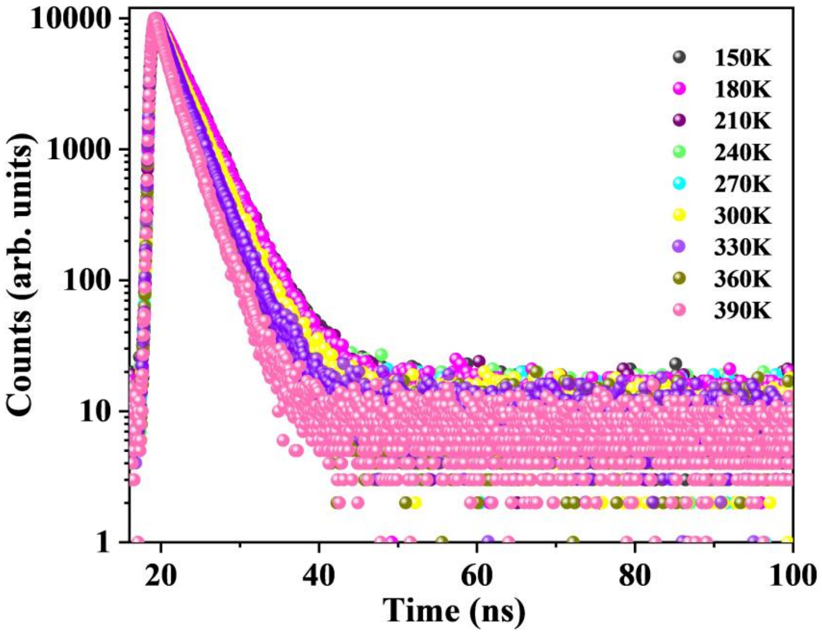

Supplementary Fig. 19. Temperature-dependent PL decay lifetimes of DAST@HPβCD fibers.

Supplementary Table 8. Summarized temperature-dependent PL decay lifetimes of DAST@HPβCD fibers.

**Temperature (K)**	τ_1 **(ns)**	**A_1 (%)**	τ_2 **(ns)**	**A_2 (%)**	$\tau_{average}$ **(ns)**
**150**	3.361	100	0	0	3.361
**180**	3.342	100	0	0	3.342
**210**	3.305	100	0	0	3.305
**240**	3.257	100	0	0	3.257
**270**	3.130	100	0	0	3.130
**300**	2.999	100	0	0	2.999
**330**	2.757	100	0	0	2.757
**360**	2.525	100	0	0	2.525
**390**	2.190	100	0	0	2.190

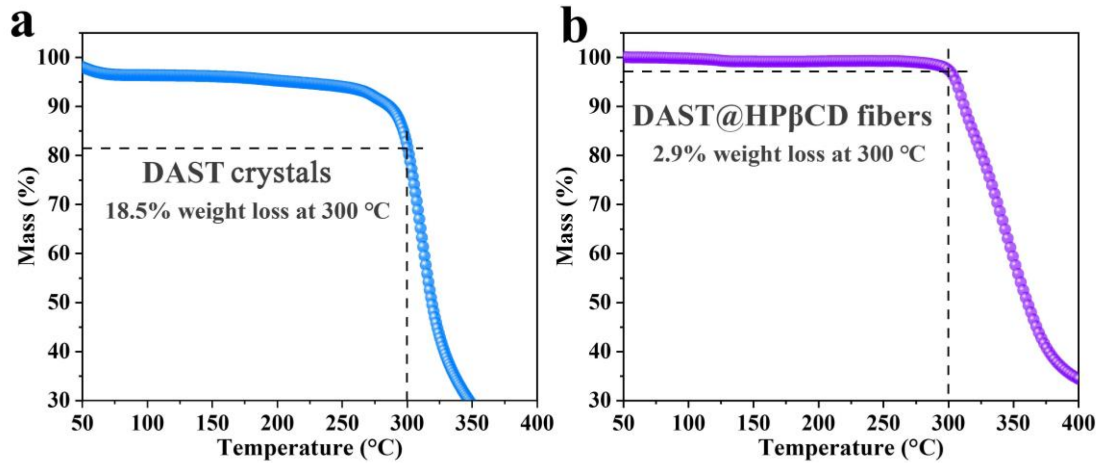

Supplementary Fig. 20. TG spectra of (a) DAST crystals and (b) DAST@HPβCD fibers.

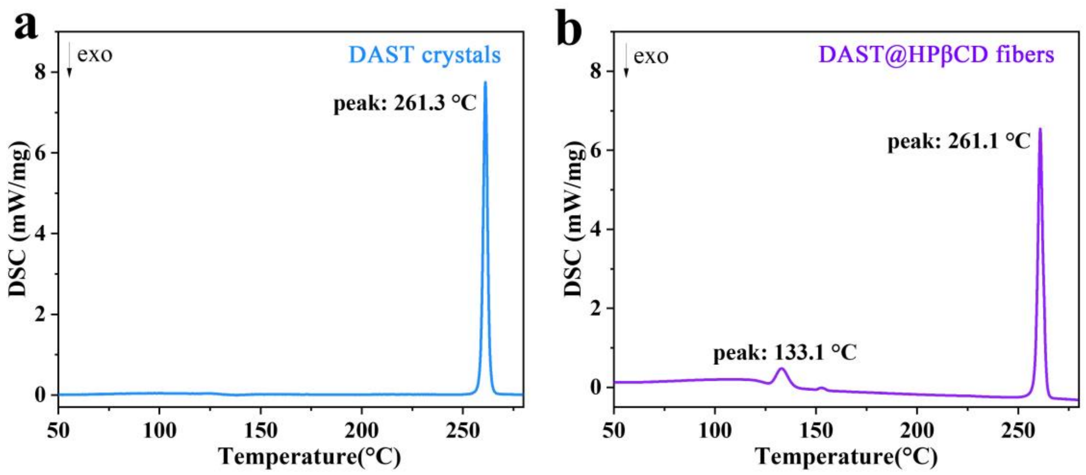

Supplementary Fig. 21. The DSC spectra of (a) DAST crystals and (b) DAST@HPβCD fibers. Before measurement, the DAST crystals were stored in N_2-filled glovebox and the DAST@HPβCD fibers were stored in ambient air with a relative humidity of 65%.

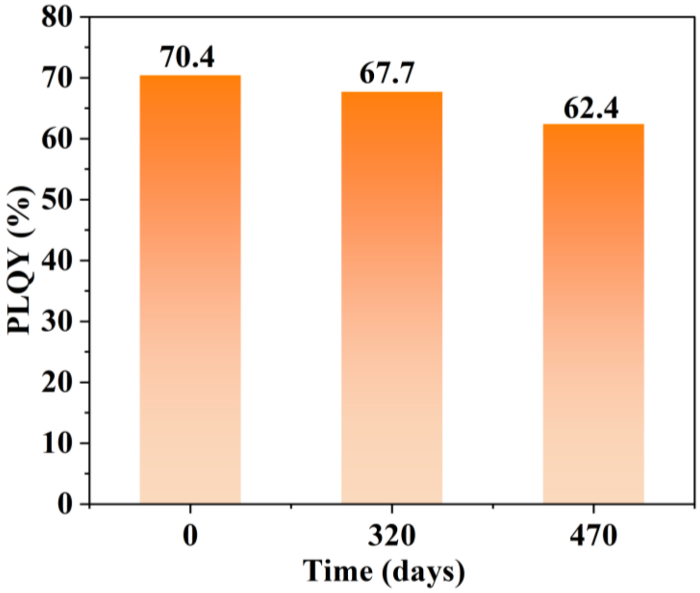

Supplementary Fig. 22. Time-dependent PLQY evolution of DAST@HPβCD fibers stored in ambient air.

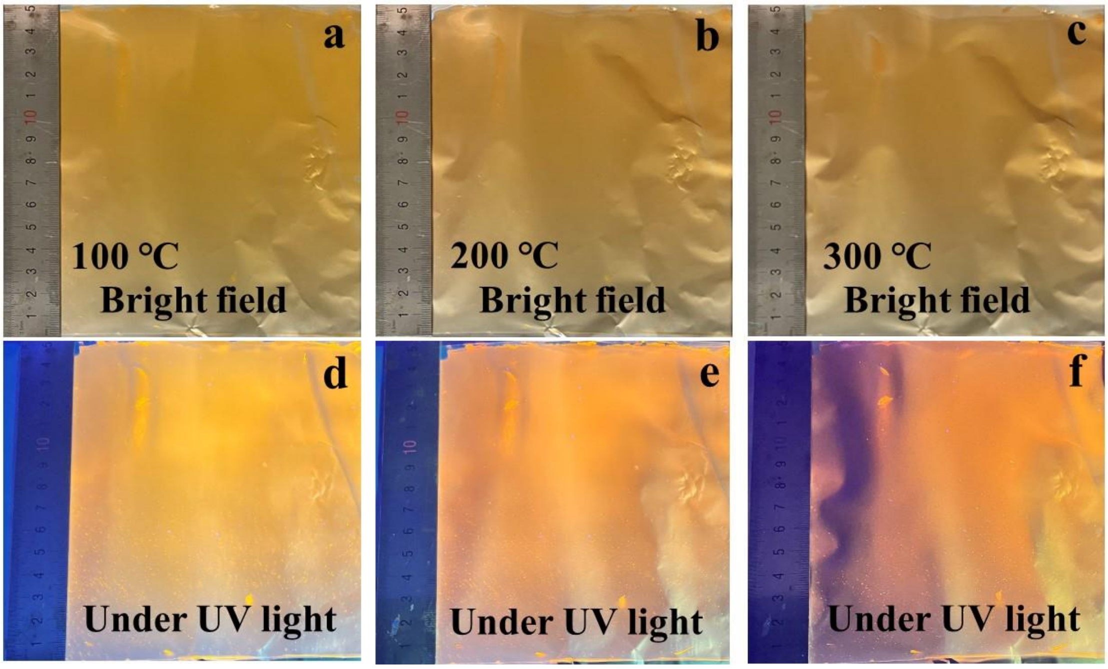

Supplementary Fig. 23. The photographs under bright field and fluorescence images under UV light of DAST@HPβCD thin film heated at (a, d) 100 °C, (b, e) 200 °C and (c, f) 300 °C.

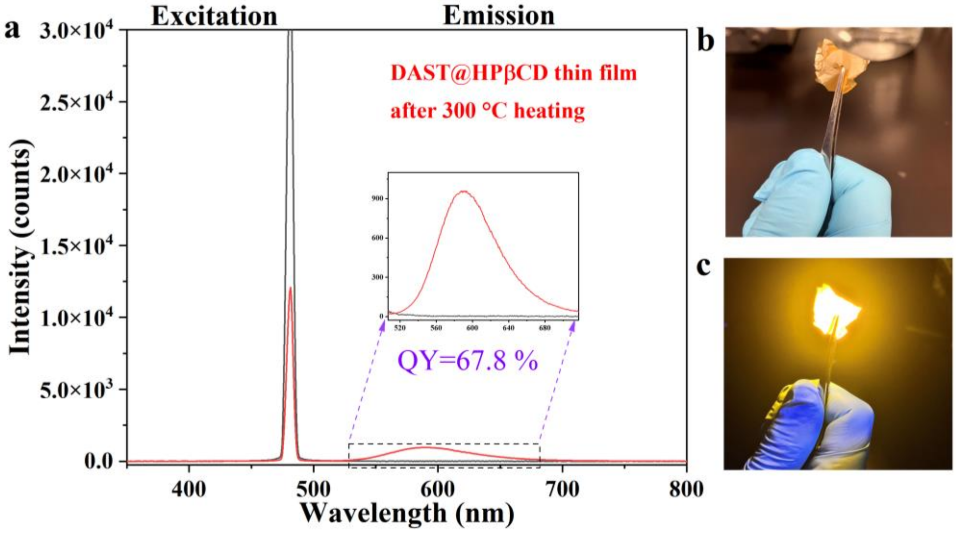

Supplementary Fig. 24. (a) The PLQY of DAST@HPβCD thin film after heating at 300 °C for 20 min. The photograph of DAST@HPβCD thin film under natural light (b) and under UV light irradiation (c).

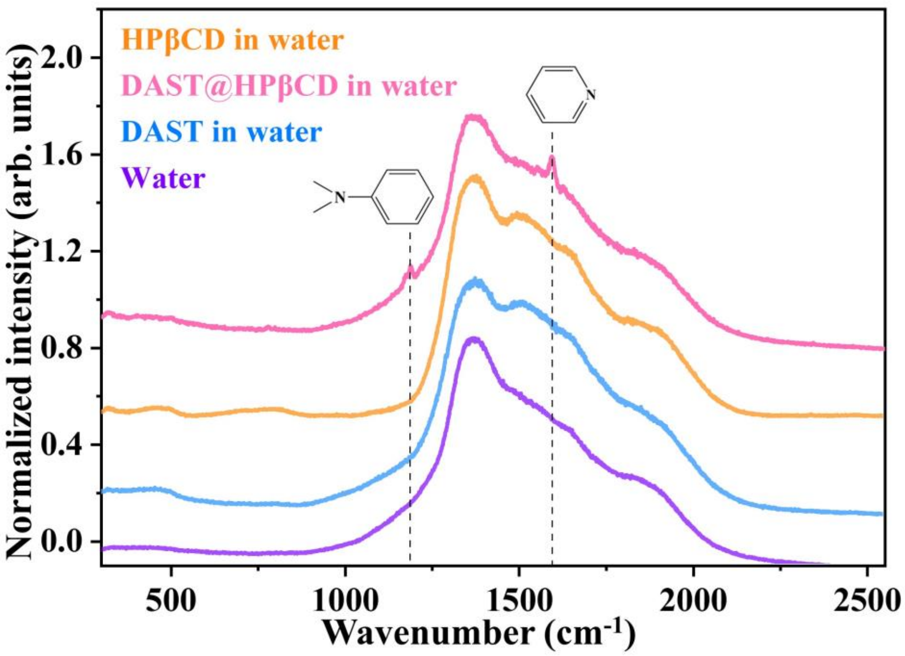

Supplementary Fig. 25. Raman spectra of water, DAST in water, HPβCD in water and DAST@HPβCD in water.

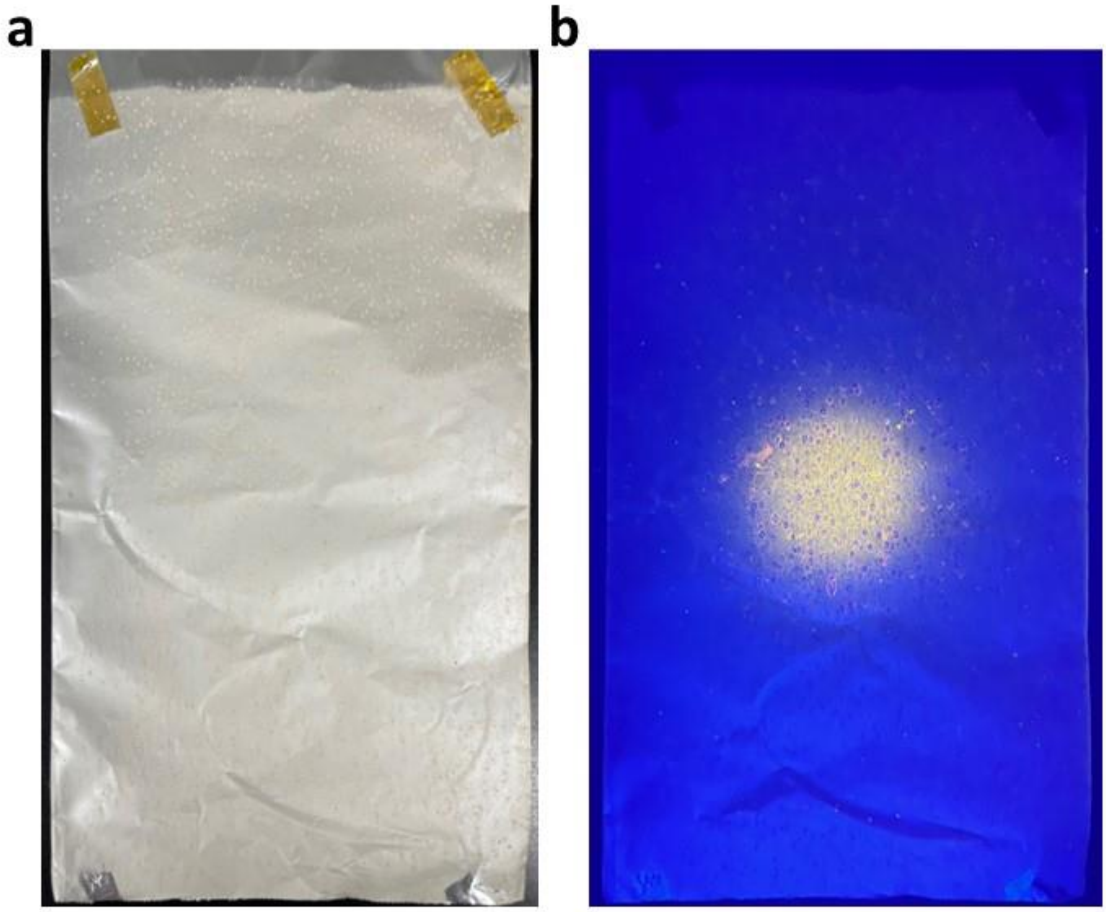

Supplementary Fig. 26. Photographs of DAST@PS fibrous membrane under natural light (a) and UV light (b).

Note, the fabrication process of DAST@PS fibers were the same as DAST@HPβCD fibers, which only changed the crosslinked HPβCD matrix to PS polymer.

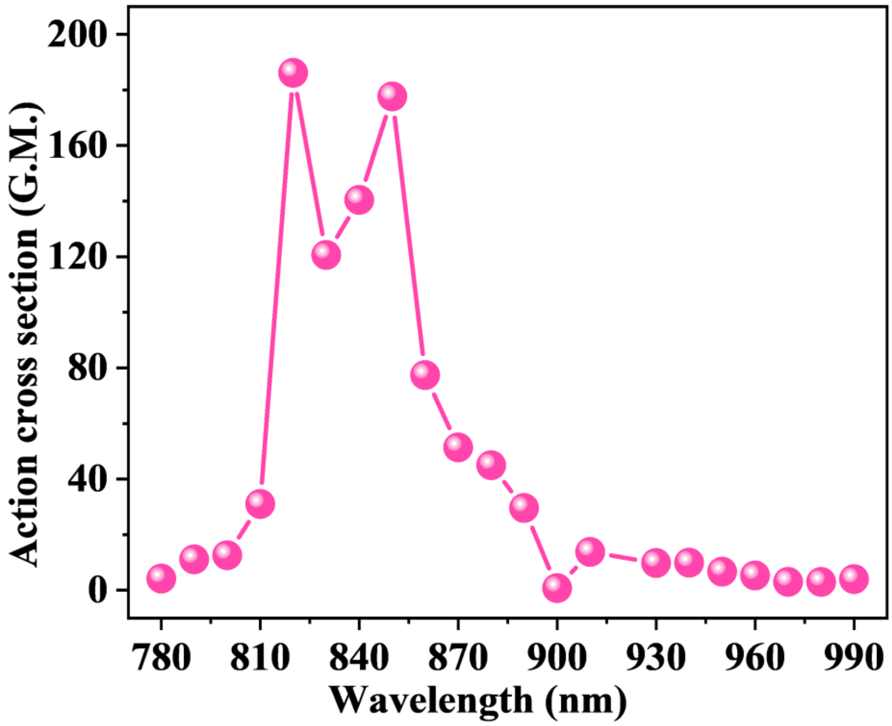

Supplementary Fig. 27. The fluctuation of two-photon action cross-section of DAST@HPβCD fibers with the change of excitation wavelength.

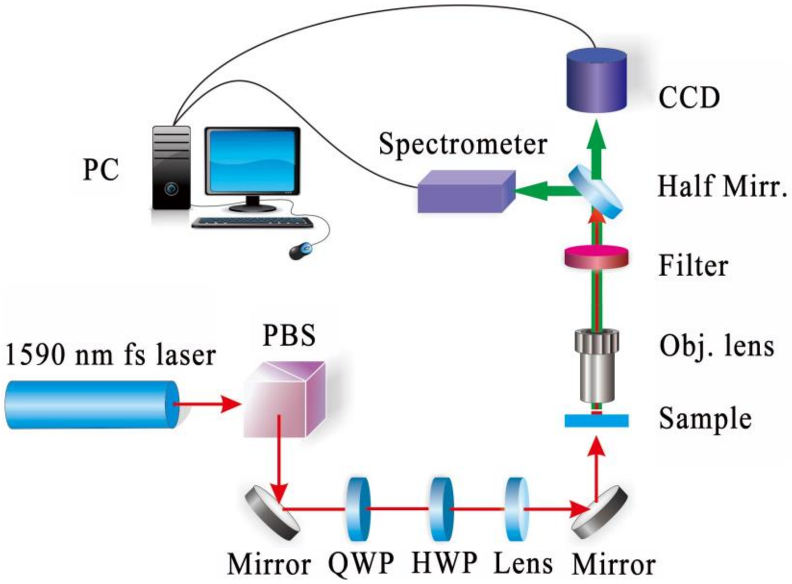

Supplementary Fig. 28. Setup of 3PEF evaluation.

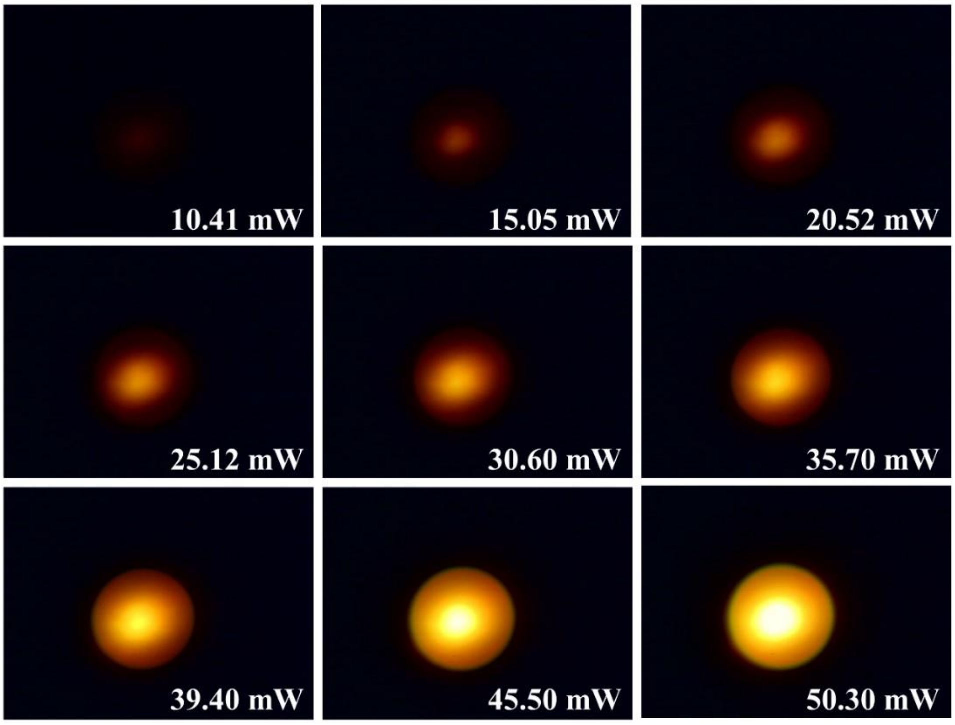

Supplementary Fig. 29. The 3PEF images of DAST@HPβCD taken at different excitation power.

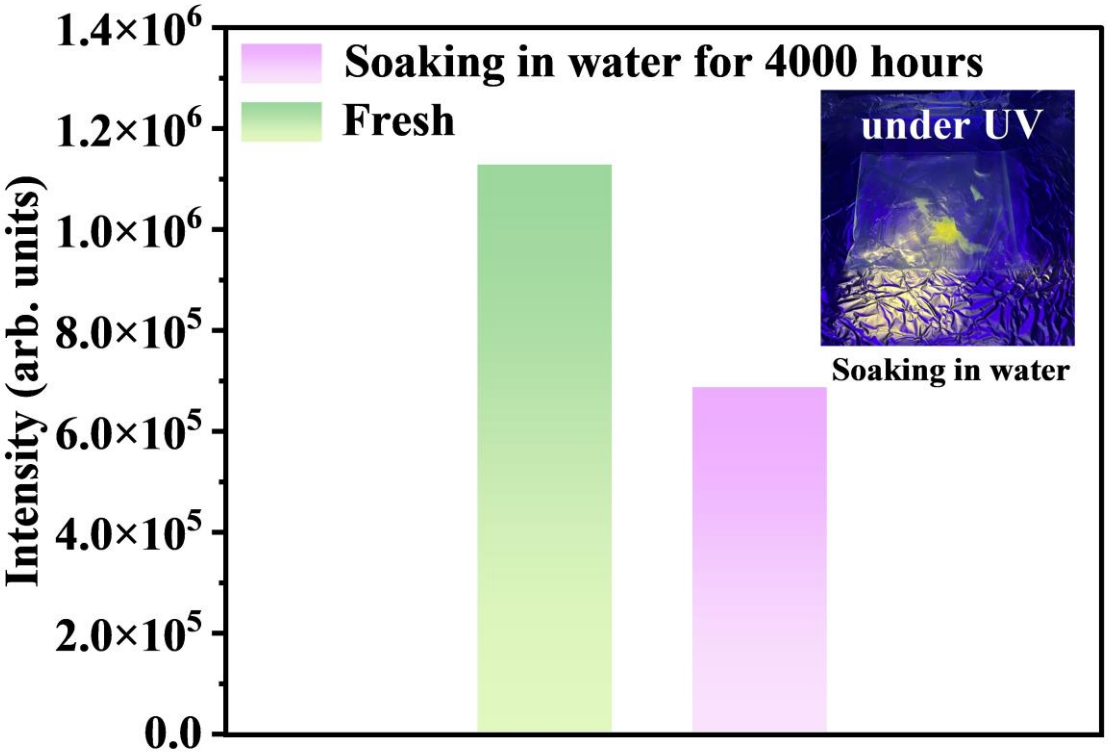

Supplementary Fig. 30. The PL intensity change of DAST@HPβCD fibers before and after being soaked in water for 4000 hours.

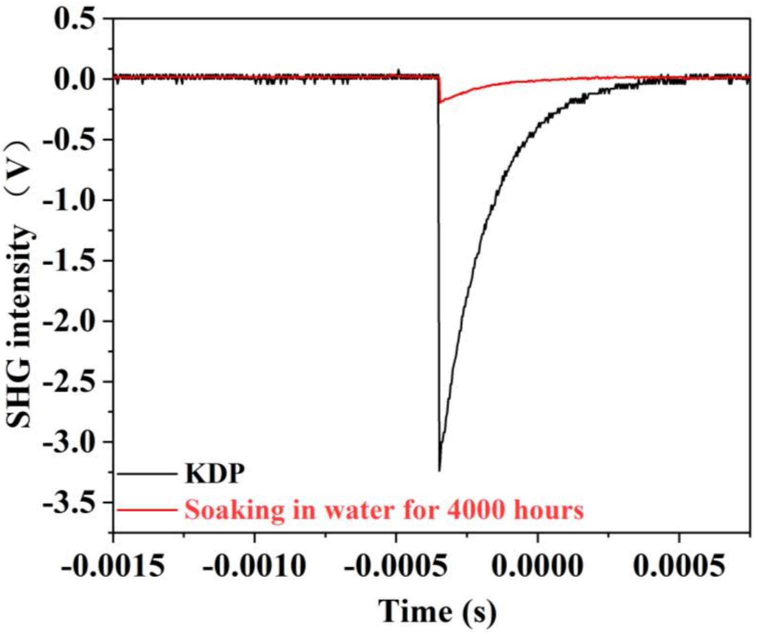

Supplementary Fig. 31. The SHG signals of the reference KDP and DAST@HPβCD fibers soaked in water for 4000 hours.

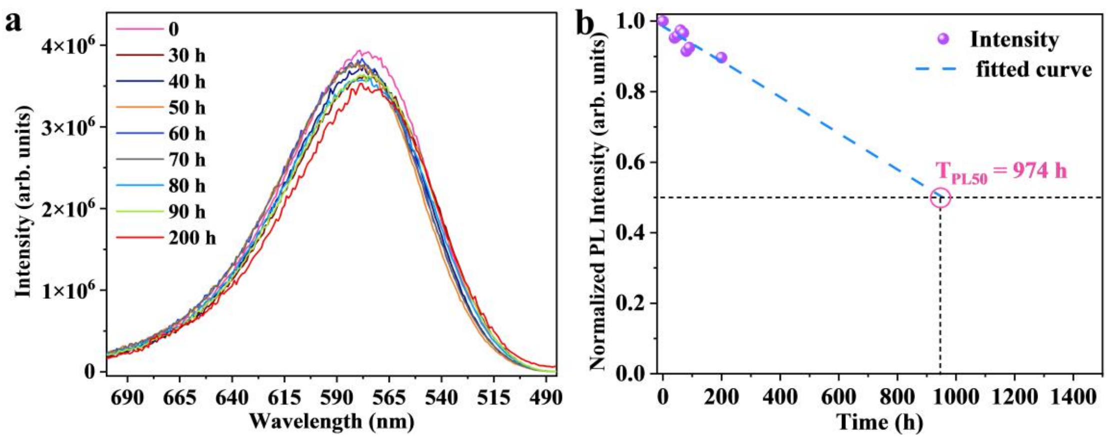

Supplementary Fig. 32. (a) Time-dependent PL intensity of the DAST@HPβCD fibers upon UV light irradiation for different durations. (b) T_{PL50} evaluation of the as-prepared DAST@HPβCD fibers under UV light irradiation.

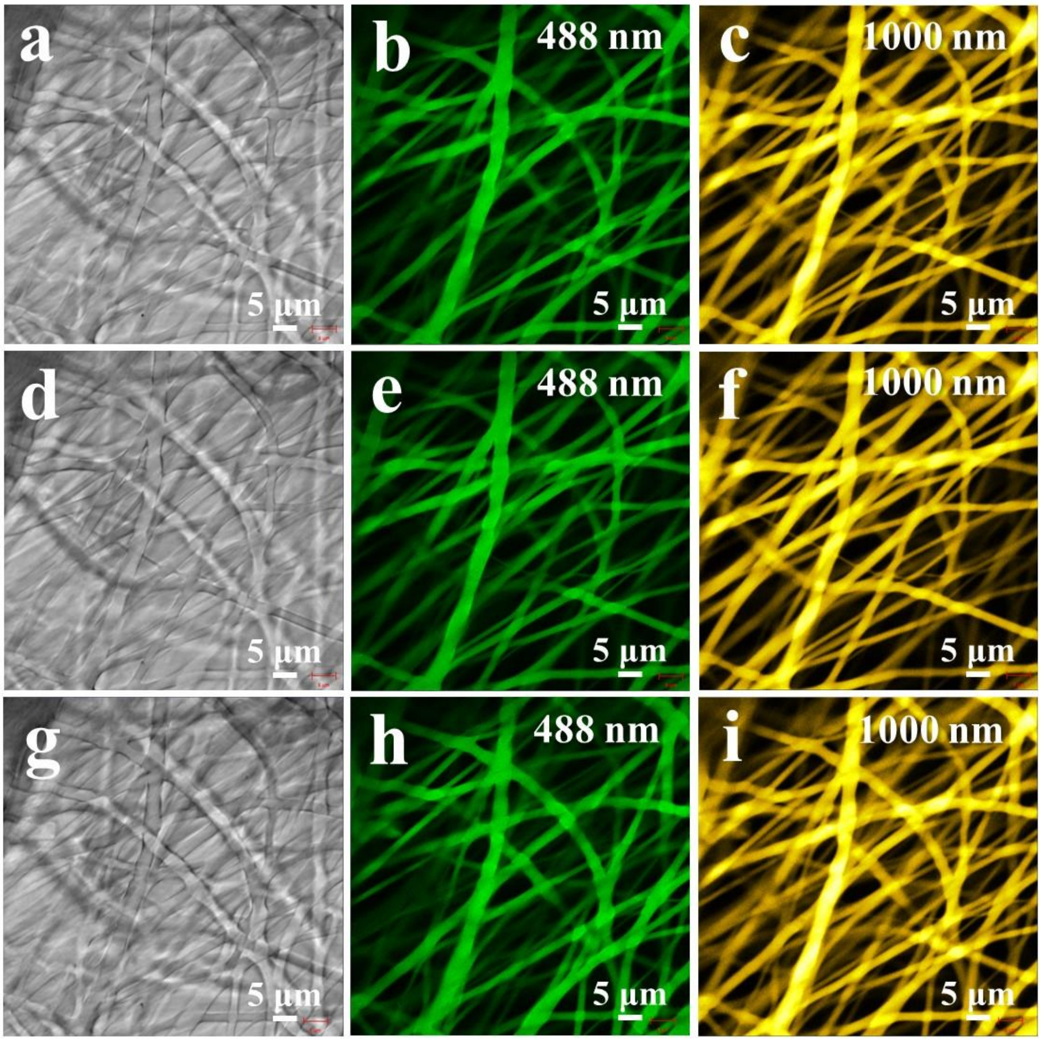

Supplementary Fig. 33. The confocal laser scanning microscopy (CLSM) images of DAST@HPβCD fibers soaked in water were taken immediately (a-c), over a period of 15 min (d-f), and over a period of 30 min (g-i), in which the images were taken at bright-field mode (left); 488 nm excitation (green color, pseudo-colored image) and 1000 nm excitation (orange color, pseudo-colored image).

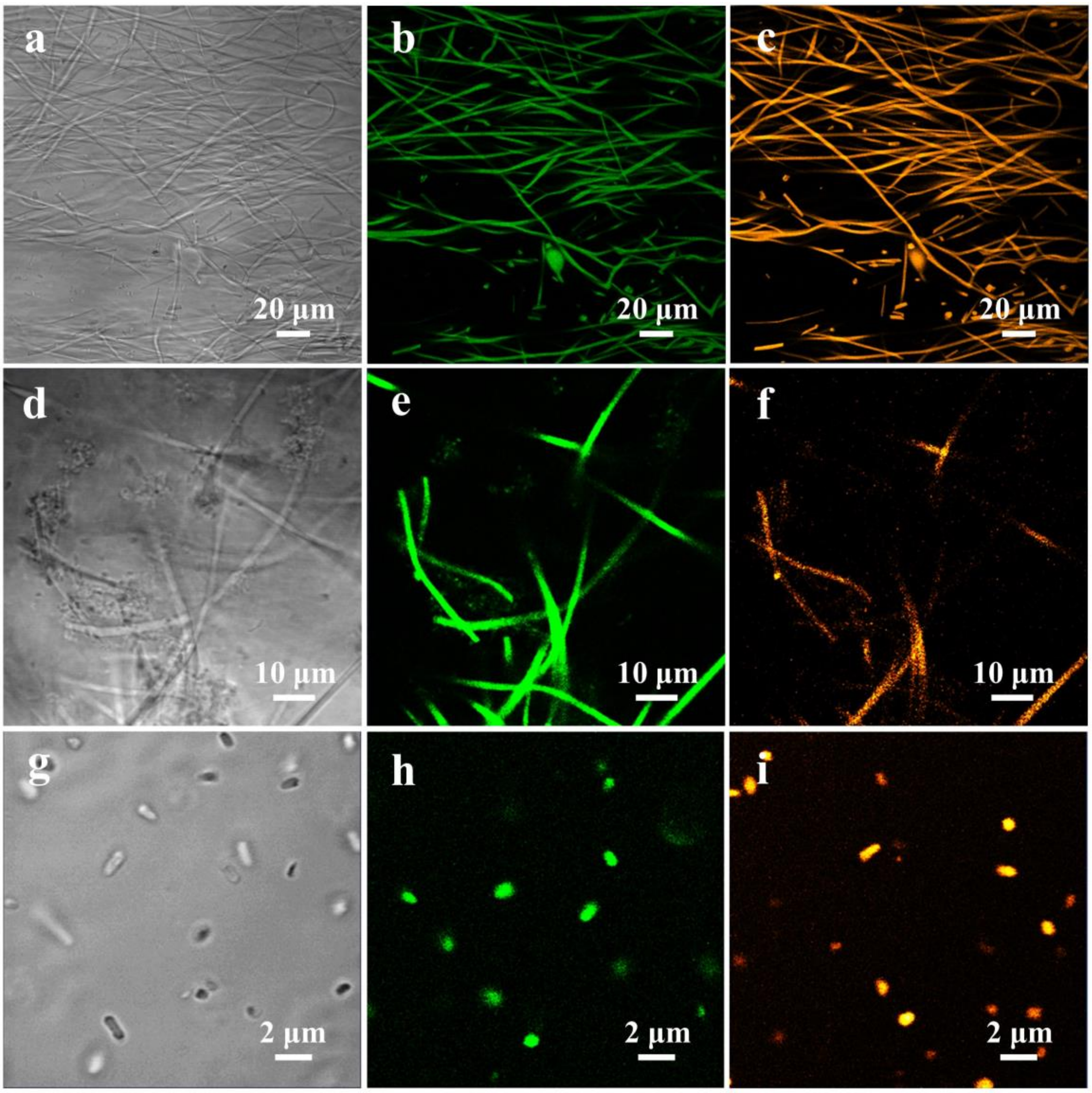

Supplementary Fig. 34. The CLSM images of *E. coli* being fed with the DAST@HPβCD fibers for 8 h (a-c); after 24 hours (d-f) and after 96 hours (g-i), in which the images were taken at bright-field mode (left); 488 nm excitation (green color, pseudo-colored image) and 1000 nm excitation (orange color, pseudo-colored image).

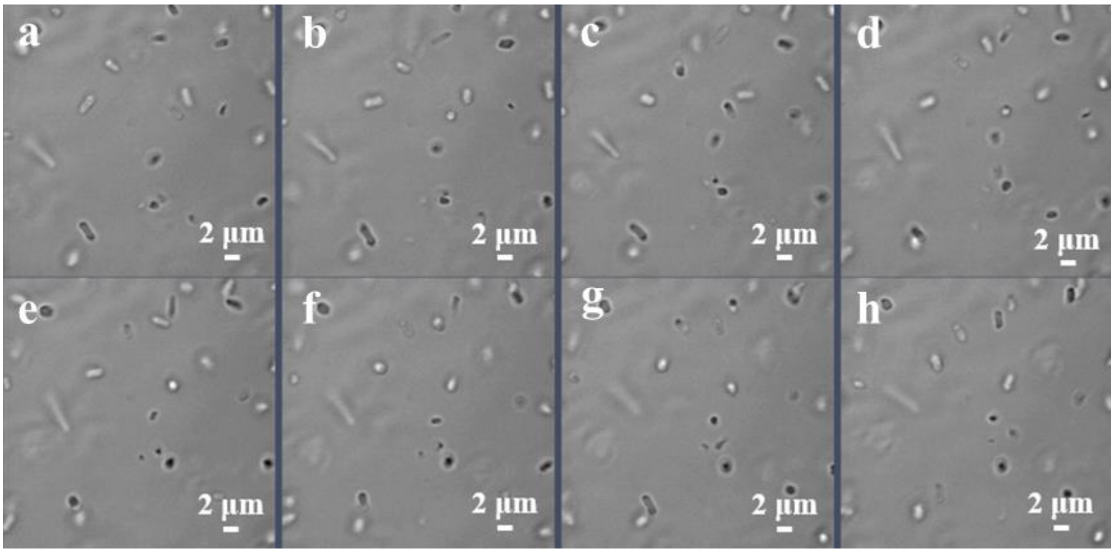

Supplementary Fig. 35. (a-h) The CLSM images of DAST@HPβCD-stained *E. coli* taken every second at bright-filed mode.

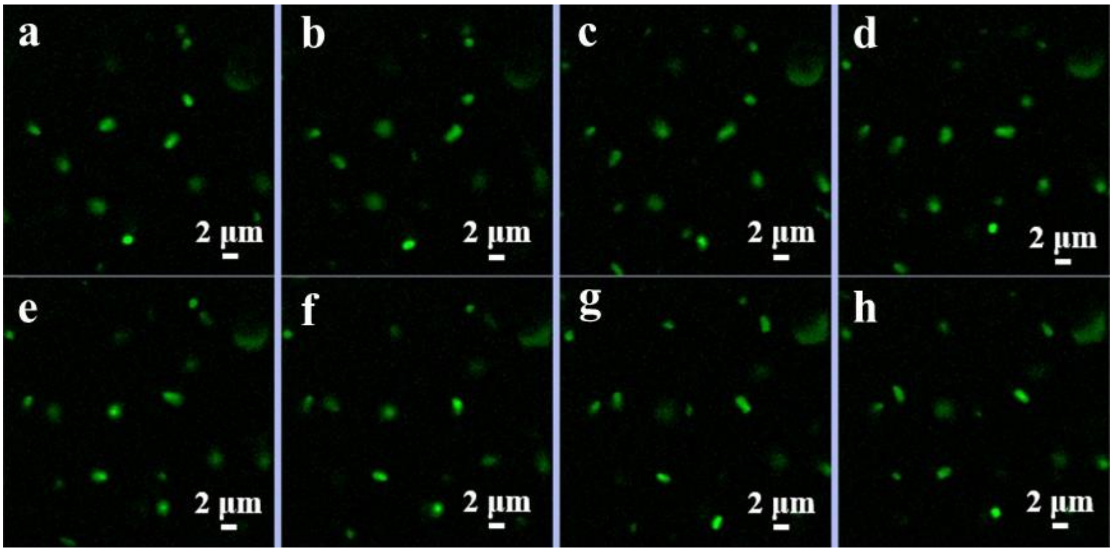

Supplementary Fig. 36. (a-h) The CLSM pseudo-colored images at 1PEF of DAST@HPβCD-stained *E. coli* taken every second at 488 nm excitation.

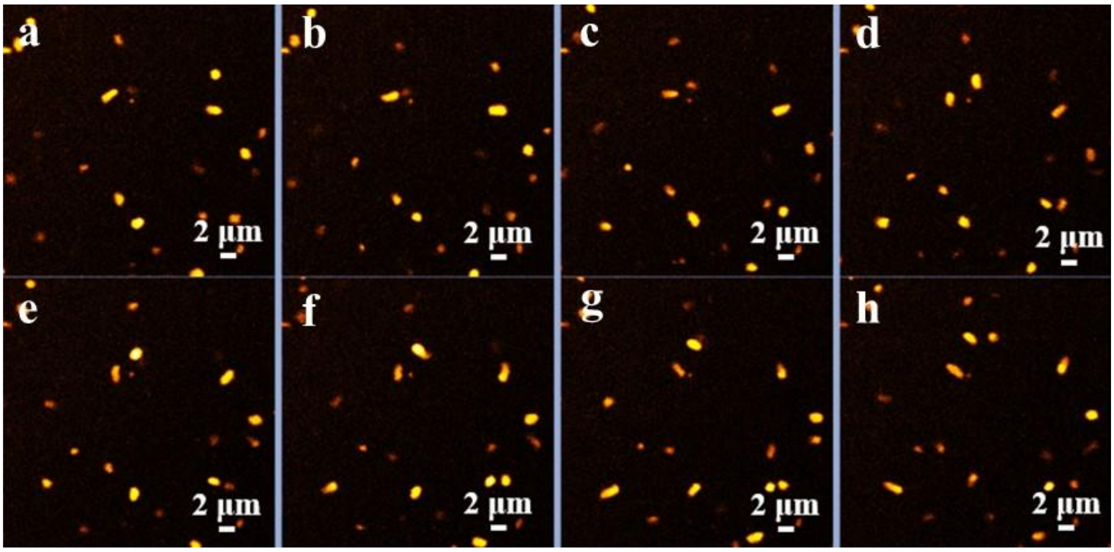

Supplementary Fig. 37. (a-h) The CLSM pseudo-colored images at 2PEF of DAST@HPβCD-stained *E. coli* taken every second at 1000 nm excitation.

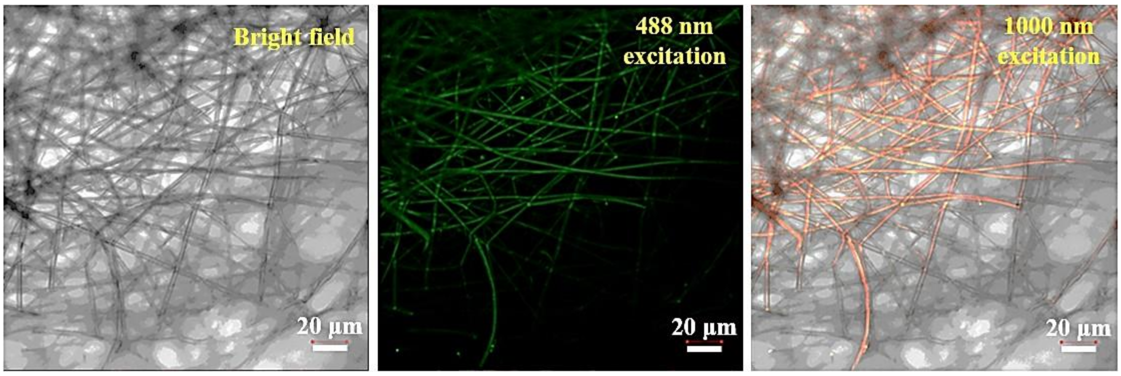

Supplementary Fig. 38. The CLSM images of DAST@HPβCD fibers immersed in culture solution without *E. coli* inoculation for 96 h, which were taken at bright-field mode (left); 488 nm excitation (green color, pseudo-colored image) and 1000 nm excitation (orange color, pseudo-colored image).

Supplementary Table 9. A summary of the state-of-the-art inorganic transition metal chalcogenides quantum dots/nanocrystals, organic fluorescence materials or organic/inorganic hybrid materials for bioimaging application.

**Composites**	**Stability**		**PLQY (%)**	**Bio-imaging application**	**Ref**
	**Water/polar solvent**	**High Temperature**			
DAST@HPβCD	Persevered ~60 % of its initial PL intensity in water for 4000 hours and continuously emitted orange fluorescence	The DAST@HPβCD membrane can continuously emit luminescence even being heated at 350 ℃	73.5	2PEF[b] in living *E. coli* **(λ_{ex} = 1000 nm)**	This work
CdSe-ZnS QDs	Remained roughly constant fluorescence intensity over a period of >9 months in water	N/A	N/A	1PEF[a] in living mouse skin **(λ_{ex} = 880 nm)**	*Ref. 3*
FeSe QDs	No notable difference in the fluorescence was detected after 5 days in deionized water and 0.1 M phosphate-buffered saline (PBS)	N/A	~40	vitro 2PEF[b] in MCF7 cells **(λ_{ex} = 800 nm)**	*Ref. 4*
ZnS: Mn NCs	Being physiologically stable for 7 days, and maintained the optical properties for 24 h in serum as well as in buffer solution	N/A	~35	3PEF[c] in living mouse skin **(λ_{ex} = 920 nm)**	*Ref. 5*
$Zn(S)_2L$ (L = N-hexyl-3-{2-[4-(2,2':6',2''-terpyridin-4'-yl)phenyl] ethenyl}carbazole	N/A	N/A	31	2PEF[b] in living HeLa cells **(λ_{ex} = 800 nm)**	*Ref. 6*

PPESO3	N/A	N/A	N/A	vitro 2PEF[b] in HeLa cells **(λ_{ex} = 750 nm)**	*Ref. 7*
TPM/TPM-Cl nanoparticles （phenyl-(2,3,4,5-tetraphenyl-1H-pyrrol-1-yl) methanone (TPM) and (4-chlorophenyl)-(2,3,4,5-etraphenyl-1H-pyrrol-1-yl) methanone (TPM-Cl)）	N/A	N/A	3.76	2PEF[b] in living HeLa cells **(λ_{ex} = 800 nm)**	*Ref. 8*
P-F8-DPSB (poly[(9,9-di-n-octylfluorene-2,7-diyl)-alt-co-(2,5-bis(4-(N,N-(diphenylmino) styryl) benzene)-1,4-diyl)]), and DPA-PR-PDI (perylenediimide derivative dye)	Exhibited size stability in PBS solution after being stored for 15 days	N/A	45	2PEF[b] in living HeLa cells **(λ_{ex} = 800 nm)**	*Ref. 9*
FL-DTE (fluorene derivative - dithienylethene)	N/A	N/A	36	N/A	*Ref. 10*

[a] One-photon excited fluorescence imaging;

[b] Two-photon excited fluorescence imaging;

[c] Three-photon excited fluorescence imaging.

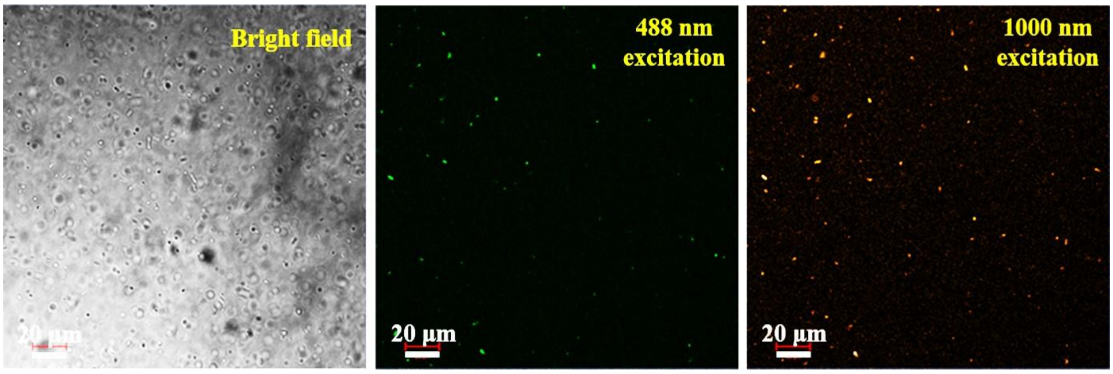

Supplementary Fig. 39. The CLMS images of DAST@HPβCD-stained *E. coli* cells which were continuously fed with LB medium for 14 days, and the images were taken at bright-filed mode (left); 488 nm excitation (green color, pseudo-colored image) and 1000 nm excitation (orange color, pseudo-colored image).

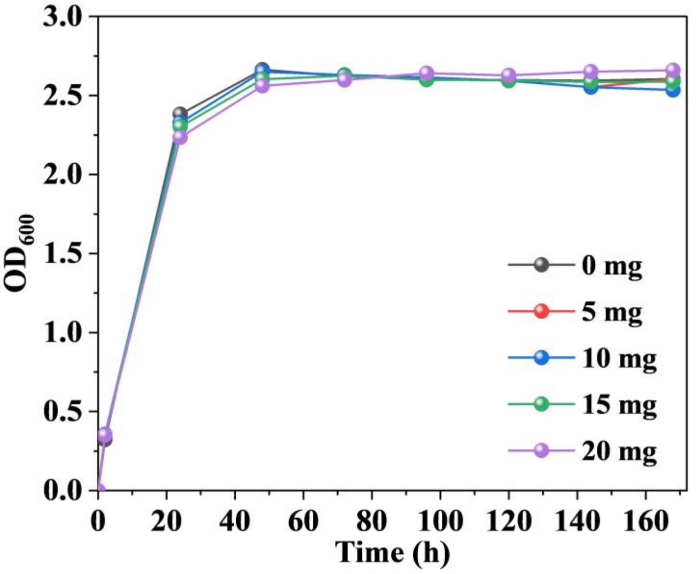

Supplementary Fig. 40. The impact of different amounts of DAST@HPβCD fibers (as indicated) on the growth performance (the change of OD_{600} over time) of *E. coli*.

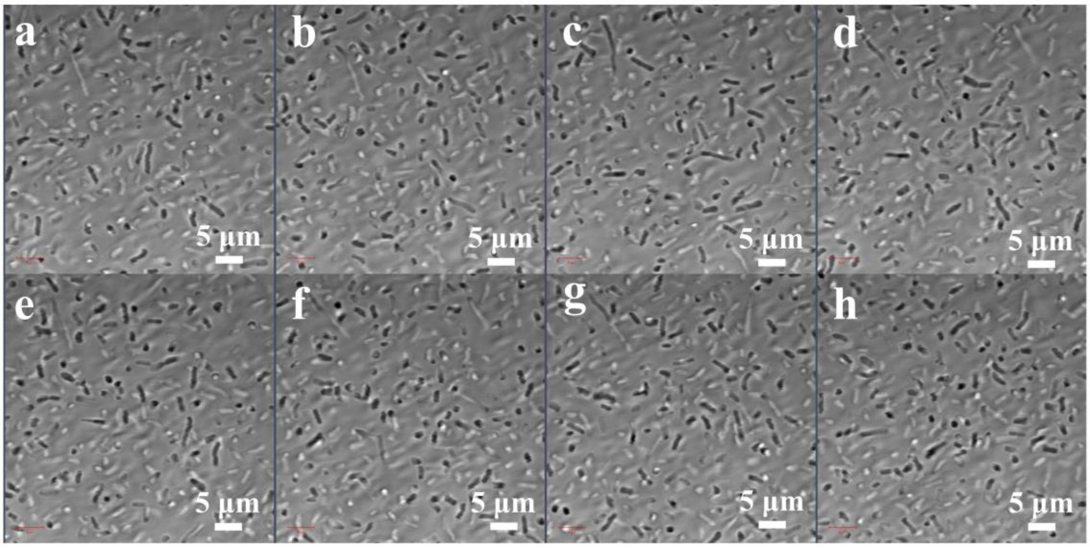

Supplementary Fig. 41. (a-h) The CLSM images of 20 mg DAST@HPβCD-stained *E. coli* taken every second using bright-filed mode.

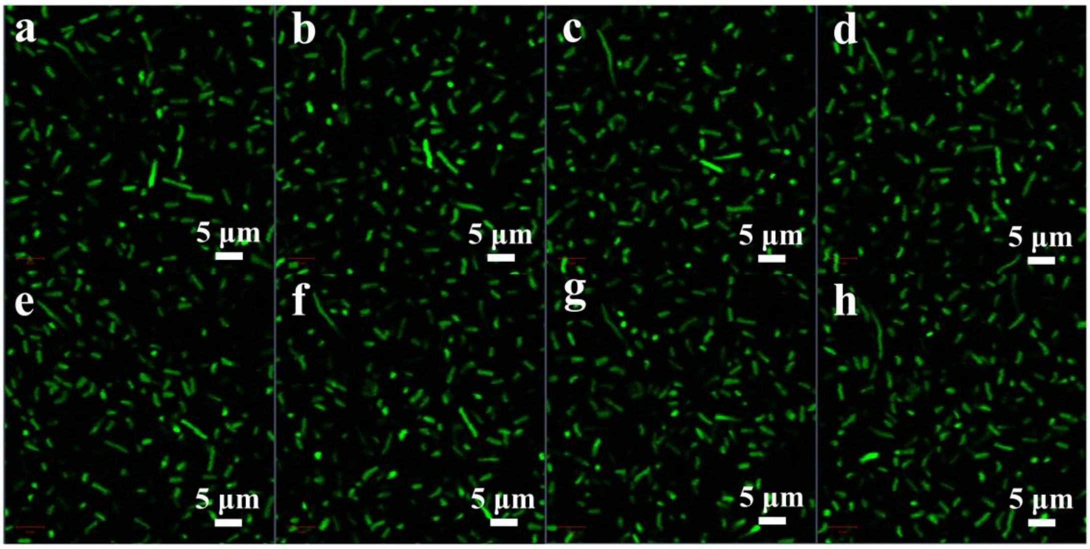

Supplementary Fig. 42. (a-h) The CLSM pseudo-colored images at 1PEF of 20 mg DAST@HPβCD-stained *E. coli* taken every second at 488 nm excitation.

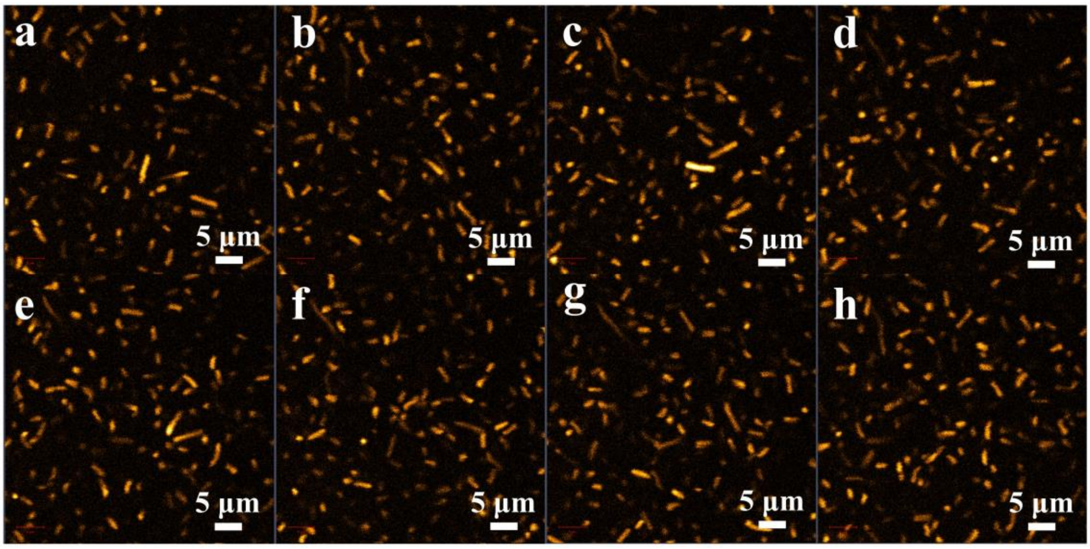

Supplementary Fig. 43. (a-h) The CLSM pseudo-colored images at 2PEF of 20 mg DAST@HPβCD-stained *E. coli* taken every second at 1000 nm excitation.

Supplementary References

1 Tian, T., Cai, B. & Sugihara, O. DAST single-nanometer crystal preparation using a substrate-supported rapid evaporation crystallization method. *Nanoscale* **8**, 18882-18886 (2016).

2 Zheng, Mei-Ling. et al, Two-photon excited fluorescence and second-harmonic generation of the DAST organic nanocrystals. *J. Phys. Chem. C.* **115**, 8988-8993 (2011).

3 Larson, D. R. et al. Water-soluble quantum dots for multiphoton fluorescence imaging in vivo. *Science* **300**, 1434-1436 (2003).

4 Kwon, J. et al. FeSe quantum dots for in vivo multiphoton biomedical imaging. *Sci. Adv.* **5**, eaay0044 (2019).

5 Yu, J. H. et al. High-resolution three-photon biomedical imaging using doped ZnS nanocrystals. *Nat. Mater.* **12**, 359-366 (2013).

6 Gao, Y. et al. A sulfur-terminal Zn(II) complex and its two-photon microscopy biological imaging application. *J. Am. Chem. Soc.* **131**, 5208-5213 (2009).

7 Parthasarathy, A. et al. Two-photon excited fluorescence of a conjugated polyelectrolyte and its application in cell imaging. *ACS Appl. Mater. Interfaces* **2**, 2744-2748 (2010).

8 Yang, J. et al. Rational design of pyrrole derivatives with aggregation-induced phosphorescence characteristics for time-resolved and two-photon luminescence imaging. *Nat. Commun.* **12**, 4883 (2021).

9 Lv, Y. et al. Conjugated polymer-based hybrid nanoparticles with two-photon excitation and near-infrared emission features for fluorescence bioimaging within the biological window. *ACS Appl. Mater. Interfaces* **7**, 20640-20648 (2015).

10 Benitez-Martin, C. et al. Toward two-photon absorbing dyes with unusually potentiated nonlinear fluorescence response. *J. Am. Chem. Soc.* **142**, 14854-14858 (2020).
