# Supplementary material for: Durable organic nonlinear optical membranes for thermotolerant lightings and in vivo bioimaging
Source: Nat Commun. 2023 Jul 22;14:4429. doi: 10.1038/s41467-023-40168-2 (PMC10363139; doi:10.1038/s41467-023-40168-2)
Supplement: Supplementary file 3 — Description of Additional Supplementary Files [file 41467_2023_40168_MOESM3_ESM.pdf]

Description of Additional Supplementary Files

File Name: Supplementary Movie 1
Description: Highspeed electrospinning process for DAST@HPβCD fibers

File Name: Supplementary Movie 2
Description: In vivo real-time imaging of fluorescent DAST@HPβCD-labled *E. Coli* at bright-field.

File Name: Supplementary Movie 3
Description: In vivo real-time imaging of fluorescent DAST@HPβCD-labled E. Coli at 488 nm excitation.

File Name: Supplementary Movie 4
Description: In vivo real-time imaging of fluorescent DAST@HPβCD-labled *E. Coli* at 1000 nm excitation.
