# Supplementary material for: Durable organic nonlinear optical membranes for thermotolerant lightings and in vivo bioimaging
Source: Nat Commun. 2023 Jul 22;14:4429. doi: 10.1038/s41467-023-40168-2 (PMC10363139; doi:10.1038/s41467-023-40168-2)
Supplement: Supplementary file 8 — Reporting Summary [file 41467_2023_40168_MOESM8_ESM.pdf]

nature portfolio

Corresponding author(s): Wu-Qiang Wu

Last updated by author(s): Jun 26, 2023

Reporting Summary

Nature Portfolio wishes to improve the reproducibility of the work that we publish. This form provides structure for consistency and transparency in reporting. For further information on Nature Portfolio policies, see our Editorial Policies and the Editorial Policy Checklist.

Statistics

For all statistical analyses, confirm that the following items are present in the figure legend, table legend, main text, or Methods section.

n/a	Confirmed	
☐	☒	The exact sample size (*n*) for each experimental group/condition, given as a discrete number and unit of measurement
☐	☒	A statement on whether measurements were taken from distinct samples or whether the same sample was measured repeatedly
☒	☐	The statistical test(s) used AND whether they are one- or two-sided<br>*Only common tests should be described solely by name; describe more complex techniques in the Methods section.*
☐	☒	A description of all covariates tested
☐	☒	A description of any assumptions or corrections, such as tests of normality and adjustment for multiple comparisons
☒	☐	A full description of the statistical parameters including central tendency (e.g. means) or other basic estimates (e.g. regression coefficient) AND variation (e.g. standard deviation) or associated estimates of uncertainty (e.g. confidence intervals)
☒	☐	For null hypothesis testing, the test statistic (e.g. *F*, *t*, *r*) with confidence intervals, effect sizes, degrees of freedom and *P* value noted<br>*Give P values as exact values whenever suitable.*
☒	☐	For Bayesian analysis, information on the choice of priors and Markov chain Monte Carlo settings
☐	☒	For hierarchical and complex designs, identification of the appropriate level for tests and full reporting of outcomes
☒	☐	Estimates of effect sizes (e.g. Cohen's *d*, Pearson's *r*), indicating how they were calculated

Our web collection on statistics for biologists contains articles on many of the points above.

Software and code

Policy information about availability of computer code

Data collection	The geometry optimizations of DAST and DAST@HPβCD were carried out by the self-consistent charge density-functional tight-binding (SCC-DFTB. release 22.2) method with a dispersion correction using the DFTB+ program. The smart algorithm with the force convergence tolerance of 0.05 kcal/mol/Å is used and the SCC tolerance is set to be 1.0×10-5 electrons. Based on the optimized geometries, single point calculation of each complex was carried out at the M06-2X/6-311+g(d,p) level of theory corrected with the Grimme's dispersion (D3) using the Gaussian 09 software package. The natural transition orbital (NTO) distributions and vertical excitation energies were calculated at the b31yp/6-31g (d) energy level.
Data analysis	To further gain insight into the frontier molecular orbital (FMO) distributions and electronic structures of the DAST and DAST@HPβCD, the natural transition orbitals (NTO) simulation was performed using the Gaussian 09 package at the M06-2X/6-311+g (d,p) (see the calculation details in the Method section of Supporting Information). It shows that the "hole" and "particle" of S1 state are dispersed on the DAST molecule (Fig. 2g), implying that, ideally, the DAST molecule could achieve LE state-dominant characteristic. However, in reality, the DAST material is not efficiently luminescent and loses its NLO properties owing to its vulnerability in ambient air and/or polar solvent environment. For DAST@HPβCD complex, the proportion of LE state increases and the oscillator strength (f) severely decreased from 1.2877 to 0.8240, which could be attributed to the increased energy gap with HPβCD inclusion.

For manuscripts utilizing custom algorithms or software that are central to the research but not yet described in published literature, software must be made available to editors and reviewers. We strongly encourage code deposition in a community repository (e.g. GitHub). See the Nature Portfolio guidelines for submitting code & software for further information.

Data

Policy information about availability of data

All manuscripts must include a data availability statement. This statement should provide the following information, where applicable:

- Accession codes, unique identifiers, or web links for publicly available datasets
- A description of any restrictions on data availability
- For clinical datasets or third party data, please ensure that the statement adheres to our policy

The source data generated in this study are provided in the 'Source Data' file. Source data are provided with this paper.

Research involving human participants, their data, or biological material

Policy information about studies with human participants or human data. See also policy information about sex, gender (identity/presentation), and sexual orientation and race, ethnicity and racism.

Reporting on sex and gender	N/A
Reporting on race, ethnicity, or other socially relevant groupings	N/A
Population characteristics	N/A
Recruitment	N/A
Ethics oversight	N/A

Note that full information on the approval of the study protocol must also be provided in the manuscript.

Field-specific reporting

Please select the one below that is the best fit for your research. If you are not sure, read the appropriate sections before making your selection.

☒ Life sciences ☐ Behavioural & social sciences ☐ Ecological, evolutionary & environmental sciences

For a reference copy of the document with all sections, see nature.com/documents/nr-reporting-summary-flat.pdf

Life sciences study design

All studies must disclose on these points even when the disclosure is negative.

Sample size	The E.Coli samples were used as received, and the sample size will not affect the analysis of bioimaging result of this work. No statistical method is used to predetermine the sample size. In this study, we conducted a bio-experiment on E. coli and evaluated its growth performance. As we employed a pure strain and observed its performance in a short term, the difference of sample sizes among the groups should not be of great significance. In accordance with previous studies on pure microbial strains and conventions, we determine the groups in triplicate, which should be considered as sufficient sample sizes in our case.
Data exclusions	There is no data exclusions in this study.
Replication	Each fluorescence labelling and bioimaging test was taken at least by twice. And all attempts at replication are successful to support the same result and conclusion.
Randomization	The bacterium was first cultivated for 24 h and then subcultured in closed serum bottles in triplicate with an inoculum size of 10%. The allocation of samples into experimental group is random. The bacterium seed for subculturing was selected randomly. And the region selected when CLSM images are taken is also random.
Blinding	The investigators were blinded to group allocation during data collection and/or analysis.

Reporting for specific materials, systems and methods

We require information from authors about some types of materials, experimental systems and methods used in many studies. Here, indicate whether each material, system or method listed is relevant to your study. If you are not sure if a list item applies to your research, read the appropriate section before selecting a response.

Materials & experimental systems

n/a	Involved in the study	
☒	☐	Antibodies
☒	☐	Eukaryotic cell lines
☒	☐	Palaeontology and archaeology
☒	☐	Animals and other organisms
☒	☐	Clinical data
☒	☐	Dual use research of concern
☒	☐	Plants

Methods

n/a	Involved in the study	
☒	☐	ChIP-seq
☒	☐	Flow cytometry
☒	☐	MRI-based neuroimaging
